# Characterization of the molecular mechanisms of silicon uptake in coccolithophores

**DOI:** 10.1111/1462-2920.16280

**Published:** 2022-11-22

**Authors:** Sarah Ratcliffe, Erin M. Meyer, Charlotte E. Walker, Michael Knight, Heather M. McNair, Paul G. Matson, Debora Iglesias‐Rodriguez, Mark Brzezinski, Gerald Langer, Aleksey Sadekov, Mervyn Greaves, Colin Brownlee, Paul Curnow, Alison R. Taylor, Glen L. Wheeler

**Affiliations:** ^1^ School of Biochemistry University of Bristol Bristol UK; ^2^ Department of Biology and Marine Biology University of North Carolina Wilmington Wilmington North Carolina USA; ^3^ Marine Biological Association, The Laboratory, Citadel Hill Plymouth UK; ^4^ School of Ocean and Earth Science University of Southampton Southampton UK; ^5^ Department of Ecology Evolution and Marine Biology and the Marine Science Institute University of California Santa Barbara California USA; ^6^ Graduate School of Oceanography University of Rhode Island Narragansett Rhode Island USA; ^7^ Environmental Sciences Division Oak Ridge National Laboratory Oak Ridge Tennessee USA; ^8^ ARC Centre of Excellence for Coral Reef Studies, Ocean Graduate School University of Western Australia Crawley Western Australia Australia; ^9^ The Godwin Laboratory for Palaeoclimate Research, Department of Earth Sciences University of Cambridge Cambridge UK

## Abstract

Coccolithophores are an important group of calcifying marine phytoplankton. Although coccolithophores are not silicified, some species exhibit a requirement for Si in the calcification process. These species also possess a novel protein (SITL) that resembles the SIT family of Si transporters found in diatoms. However, the nature of Si transport in coccolithophores is not yet known, making it difficult to determine the wider role of Si in coccolithophore biology. Here, we show that coccolithophore SITLs act as Na^+^‐coupled Si transporters when expressed in heterologous systems and exhibit similar characteristics to diatom SITs. We find that *CbSITL* from *Coccolithus braarudii* is transcriptionally regulated by Si availability and is expressed in environmental coccolithophore populations. However, the Si requirement of *C. braarudii* and other coccolithophores is very low, with transport rates of exogenous Si below the level of detection in sensitive assays of Si transport. As coccoliths contain only low levels of Si, we propose that Si acts to support the calcification process, rather than forming a structural component of the coccolith itself. Si is therefore acting as a micronutrient in coccolithophores and natural populations are only likely to experience Si limitation in circumstances where dissolved silicon (DSi) is depleted to extreme levels.

## INTRODUCTION

The coccolithophores are a group of calcifying marine phytoplankton belonging to the Haptophyta. Coccolithophores are characterized by their extracellular covering of calcium carbonate plates known as coccoliths, which are produced intracellularly within a membrane‐bound vesicle and secreted to the cell surface (Taylor et al., 2017). Calcification by coccolithophores contributes significantly to global carbon cycling, with the sinking of coccoliths representing a major flux of inorganic carbon to the deep oceans (Skampa et al., 2020; Ziveri et al., 2007). As coccolithophores generate their biomineralized structures from calcium carbonate, it is commonly thought that they possess a competitive advantage over silicified biomineralized phytoplankton, primarily represented by the diatoms, in regions where dissolved silicon (DSi) is potentially limiting for diatom growth (Leblanc et al., 2009). However, we recently identified that certain coccolithophore lineages exhibit a requirement for Si in the calcification process, suggesting that Si limitation could also influence the biogeography of some coccolithophore species (Durak et al., 2016). We must therefore establish the nature of the Si requirement in coccolithophores in order to understand how it may influence their physiology and ecological interactions with heavily silicified phytoplankton lineages.

Treatment of the coccolithophores *Scyphosphaera apsteinii*, *Coccolithus braarudii*, *Syracosphaera pulchra*, and *Calcidiscus leptoporus* with the Si analogue germanium (Ge) leads to significant disruption of calcification (Durak et al., 2016; Langer et al., 2021). Ge appears to specifically disrupt morphogenesis of the developing calcite crystal, rather than the ability to precipitate calcium carbonate, suggesting that Si is required for the generation of correct crystal morphology (Langer et al., 2021). In support of this hypothesis, production of holococcoliths by the haploid life cycle phase of these species is not disrupted by Ge (Langer et al., 2021). Holococcoliths consist of simple rhombic crystals and do not require the specialized crystal morphology necessary for heterococcolith formation. All further references to calcification in the manuscript will refer specifically to formation of heterococcoliths, unless stated otherwise. The requirement for Si is not found in all coccolithophores, as growth and calcification in several species belonging to the Pleurochrysidaceae and the Noelaerhabdaceae, including the major bloom forming species *Emiliania huxleyi*, are insensitive to Ge (Durak et al., 2016; Langer et al., 2021). *Emiliania huxleyi* commonly blooms in Si‐depleted stratified surface waters following a diatom bloom (Leblanc et al., 2009; Silkin et al., 2020), suggesting that competitive interactions with silicified phytoplankton may have provided selective pressure to lose their Si requirement. Assuming that the role of Si in calcification represents the ancestral state, these phylogenetic relationships indicate that the requirement has been lost at least twice in coccolithophore evolution (Durak et al., 2016).

To better understand the competitive interactions between coccolithophores and other phytoplankton, we must gain a deeper understanding of the mechanisms of Si uptake from the environment and its internal biochemistry in coccolithophores. The cellular Si quota of coccolithophores is likely to be substantially lower than heavily silicified phytoplankton, although some non‐silicified organisms, such as the unicellular cyanobacterium *Synechococcus*, can accumulate substantial intracellular concentrations of non‐mineralized Si (Baines et al., 2012; Brzezinski et al., 2017; Ohnemus et al., 2018). Heavily silicified organisms require micromolar concentrations of DSi for maximum growth in laboratory culture, whereas coccolithophores are typically grown in seawater media without Si amendments. Nevertheless, coccolithophores can experience Si limitation, as growth of *C. braarudii* or *S. pulchra* in very low DSi (<0.2 μM) resulted in coccolith malformations that were highly similar to those observed after treatment with Ge (Langer et al., 2021; Walker et al., 2018). Surface ocean DSi concentrations are typically <10 μM and reach 0.5–1.5 μM in the extensive subtropical gyres, although in Antarctic regions concentrations in excess of 50 μM are common (Treguer, 2014). Drawdown of DSi by silicifiers has a major impact on the global silica cycle and can result in very low surface ocean DSi concentrations (Yool & Tyrrell, 2003). For example, surface ocean DSi in the Western English Channel is around 5 μM during the winter when the water column is well mixed, but in summer stratified waters following the diatom spring bloom DSi is often lowered to the sub‐micromolar range (Smyth et al., 2010). A better understanding of Si uptake and cellular quotas in coccolithophores will help us understand whether they could experience DSi concentrations that are potentially limiting for growth and/or calcification in natural environments.

The ability of diatoms to substantially deplete DSi from the surrounding seawater is due to the presence of a family of Na^+^‐coupled Si transporters known as SITs that are responsible for high‐affinity Si uptake (Durkin et al., 2016; Hildebrand et al., 1997; Thamatrakoln & Hildebrand, 2007). SITs have 10 transmembrane domains (10‐TM), arranged in an inverted‐repeat topology consisting of two 5‐TM domains (Thamatrakoln et al., 2006). The SITs were first discovered in diatoms but were later found to be present in other silicifying organisms, such as choanoflagellates and chrysophytes (Likhoshway et al., 2006; Marron et al., 2013). More recently, we identified that a SIT homologue was also present in the silicifying haptophyte, *Prymnesium neolepis*, and we also identified a 10‐TM SIT in a non‐silicifying haptophyte, the calcifying coccolithophore *S. apsteinii* (Durak et al., 2016). In addition to 10‐TM SITs, a family of related proteins known as SITLs was identified in coccolithophores. The SITLs possess 5‐TM domains and show significant similarity to the individual N‐ and C‐terminal 5‐TM regions of SITs. SITLs are found in all coccolithophores that have a Si requirement but were absent from lineages that were insensitive to Ge (Durak et al., 2016). The novel SITLs are likely candidates to support high‐affinity Si uptake by coccolithophores, as all other known classes of active eukaryote Si transporters appear absent from the Si‐requiring coccolithophores (Marron et al., 2016; Ratcliffe et al., 2017). SITLs have also been found in diverse eukaryote lineages (both silicified and non‐silicified) including foraminifera, metazoa, dinoflagellates, and radiolarians (Durak et al., 2016; Marron et al., 2016), suggesting that SITLs may represent a widespread mechanism for Si transport in eukaryotes. However, SITLs have not yet been characterized experimentally. Whilst the presence of SITLs in heavily silicified lineages (e.g. radiolarians) supports the hypothesis that this novel family of proteins could also act as Si transporters, there is currently no evidence for their ability to transport Si, other than their similarity to the individual N‐ and C‐terminal 5‐TM regions of 10‐TM SITs.

Despite the clear requirement for Si in coccolithophore calcification, we currently have very little information on the nature of Si utilization by coccolithophores and its influence on their physiology and biogeography. We have therefore attempted to address the following key questions: Do the novel SITL proteins found in coccolithophores act as Si transporters? Is their gene expression regulated by Si availability? Can we detect Si uptake by coccolithophores directly? What are the cellular Si quotas of coccolithophore species that exhibit differing requirements for Si? We find that coccolithophore SITLs act as Na^+^‐coupled Si transporters in heterologous systems and native SITLs exhibit transcriptional regulation in response to changes in Si availability. However, we do not find evidence for significant rates of Si transport of exogenous Si by coccolithophores, either through drawdown of DSi or by uptake of the radiotracer ^32^Si(OH)_4_. We show that the cellular Si quotas are very low, with only trace amounts present in the coccoliths, suggesting that the rates of exogenous Si uptake required by coccolithophores are likely to be much lower than the detection limits of the existing methods for measuring Si transport.

## EXPERIMENTAL PROCEDURES

### Phytoplankton strains and culture conditions

Coccolithophores, *C. braarudii* (PLY182g + HOL), *C. leptoporus* (HET + HOL), *S. apsteinii* (RCC 1456 HET), *E. huxleyi* (CCMP1516 and B92/11), *G. oceanica* (RCC 1303HET), *S. pulchra* (HOL), *Calytrosphaera* sp. (HOL), the silicifying haptophyte *Prymnesium neolepis* (RCC 3432), and diatom *Thalassiosira weissflogii* (PLY541) cultures were grown in filtered seawater (FSW) with added f/2 nutrients (Guillard, 1975). Cells were grown in 40 ml batch cultures of autoclaved and filter sterilized Gulf Stream seawater, supplemented with either F/2 (*T. weissflogii*, *P. neolepis*, *E. huxleyi*, and *C. braarudii*) or LH nutrients and vitamins (*G. oceanica*, *C. leptoporus*, and *S. apsteinii*). LH media used was modified from the Tomas Lab (Fowler et al., 2015) which itself is modified L1. All cultures were maintained at 15°C on a 14:10 h light: dark cycle, at approximately 100 μmol m^−2^ s^−1^. The FSW was collected in May from the Western English Channel, after the diatom spring bloom, to ensure naturally low [DSi] concentrations (≤2 μM). Where FSW with very low [DSi] was required (<2 μM), the diatom *T. weissflogii* (PLY541) was used to further deplete the [DSi] as described previously (Durak et al., 2016). The diatom‐depleted seawater was used with the addition of f/4 nutrients (without Si).

### Cloning of CbSITL from C. braarudii

*CbSITL* was previously identified in the MMETSP database (CAMNT_0025525031) (Durak et al., 2016; Keeling et al., 2014). We confirmed the nucleotide sequence of *CbSITL* was correct using RT‐PCR from *C. braarudii* (PLY182g) followed by DNA sequencing. A synthetic gene for the *CbSITL* open reading frame was generated (ON982215) using codon optimization for expression in *S. cerevisiae* (DNA 2.0, ATUM, Newark, CA) and subcloned into the expression plasmid pYES2/CT (ThermoFisher, Inchinnan, UK) with the stop codon omitted, to allow the addition of a V5 epitope and a polyhistidine tag at the C‐terminus.

### Expression of *CbSITL* in *Xenopus* oocytes

cDNA was generated from the synthetic *CbSITL* construct. *CbSITL* mRNA was prepared with the mMessage mMachine T7 kit (ThermoFisher) using linearized cDNA. For each experiment, 50 *Xenopus laevis* oocytes were each injected with 10 ng RNA in 14 nl volume. After 3 days, protein expression was confirmed by Western blotting against the V5 epitope at the C‐terminus. *Xenopus* oocytes were placed into 0.5 ml of ND96 media containing 100 μM DSi and the rate of silicic acid uptake was measured by determining the concentration of DSi at specific time points using a molybdenum blue assay (Kirkwood, 1989). Oocytes injected with water were used as the control. Both control and test samples showed a small, rapid initial decrease in DSi concentration over the first 15 min that we have interpreted as Si adsorption to surfaces and not included in the calculation of Si transport rates.

### Recombinant expression of *CbSITL* in yeast

The *CbSITL* construct was expressed in the protease‐deficient *S. cerevisae* strain FGY217 (MATα, ura3‐52, lys2Δ201, pep4Δ) and then purified in a manner described by Knight et al. (2016). Briefly, yeast cells were harvested, lysed and cell membranes were sedimented after centrifugation at 180,000*g* for 1 h. Membranes were resuspended in 50 mM TrisHCl, pH 7.4, 150 mM NaCl, 5% glycerol and homogenized. Proteins were solubilized using 2% FC‐12 and CbSITL was then applied to a 1 ml ‘HisTrap’ Ni2+ column (GE Healthcare, Chalfont St Giles, UK) equilibrated in column buffer (50 mM TrisHCl, pH 7.4, 150  M NaCl, 5% glycerol, + 0.1% FC‐12) plus 20 mM imidazole. The column was washed with 40 ml of Column Buffer plus 60 mM imidazole, and then CbSITL was eluted in Column Buffer with 0.5 M imidazole. Imidazole was immediately removed by gel filtration. Recombinant CbSITL was used to prepare proteoliposomes.

### Measurement of Si transport in proteoliposomes

Liposomes were prepared from *E. coli* polar lipids as described by Knight et al. (2016). Proteoliposomes were reconstituted by combining 500 μl of extruded liposomes with 45 μl of 20% sodium cholate and 55 μl purified recombinant CbSITL at 360:1 (w/w) lipid: protein ratio for 30 min at room temperature. Cholate was removed by gel filtration (PD SpinTrap G‐25 column, Merck, Gillingham, UK). The eluted proteoliposomes were centrifuged at 200,000*g* for 1 h. For influx assays, the pellet was resuspended at 40 mg ml^−1^ in 50 mM Tris, 150 mM KCl, pH 7.4. Silicic acid was either prepared by incubating 4 ml of 0.2 M sodium silicate with 1.5 g of acidified Dowex 50WX4–50 cation exchange resin. This preparation was confirmed as monomeric silicic acid by studying the kinetics of the reaction with ammonium molybdate to form the yellow silicomolybdate product (Kirkwood, 1989). Silicic acid concentrations were subsequently verified by silicomolybdate assays and ICP‐MS. For kinetic influx measurements, proteoliposomes in 50 mM Tris, 150 mM KCl, pH 7.4 were loaded with 6 mM Zn acetate by two cycles of freeze–thaw and treated with 1 μM valinomycin. A stopped‐flow instrument (Applied Photophysics, Leatherhead, UK) was configured so that each reaction comprised one volume proteoliposomes and 10 volumes of 50 mM Tris, 150 mM NaCl, pH 7.4 plus silicic acid as required. Fluorescence excitation was at 254 nm with a 360 nm cut‐off filter. The change in apparent fluorescence due to the formation of zinc silicate complexes was monitored over time (10 s) (Knight et al., 2016). This probably represents a change in sample second‐order scattering rather than a specific fluorescence signal per se, but the signal change is detected in the fluorescence channel of the stopped‐flow instrument and so the term ‘apparent fluorescence’ is appropriate.

### qRT‐PCR of *CbSITL* expression in *C. braarudii*

For RNA extractions, 20 ml of exponential growth phase cultures (approximately 20,000 cells ml^−1^) were harvested by centrifugation at 3800*g* for 5 min at 4°C. Total RNA was extracted using Isolate II RNA Mini Kit (Bioline, Meridian Bioscience, London, UK). Extractions were treated with RQ1 RNase‐free DNase (Qiagen, Manchester UK), checked for purity using a Nanodrop 1000 (ThermoFisher) (A_260_/A_280_ ratios > 1.80) and RNA quantified using Quantifluor Single‐tube RNA System (Promega, Chilworth, UK) and a 100 ng μl^−1^ standard. The 50 ng of cDNA was synthesized per sample/standard using a SensiFAST cDNA Synthesis Kit (Bioline) with additional no Reverse‐transcriptase controls (NRTCs) for each treatment to ensure no genomic DNA contamination had occurred. Primers were designed using Geneious R8 (Biomatters Ltd, Auckland, NZ) to *CbSITL* and the reference genes *EFL* and *RPS1* to generate products approximately 150 bp in length (Table S1).

qRT‐PCR reactions were conducted using a Rotorgene 6000 cycle (Qiagen, USA) in 10 or 20 μl reaction volumes of SensiFAST No‐ROX Kit (Bioline, UK). PCR reactions were conducted with 400 nM final primer concentration for *EFL* and 200 nM final concentrations for *SITL* and *RPS1* with the following settings: initial 95°C 2 min hold, followed by 40 cycles of 95°C denaturing for 5 s, 62°C annealing for 10 s and 72°C extension step (acquisition at end of extension step) for 20 s. A high‐resolution melt (HRM) curve, 72–95°C with 1°C ramp was conducted after amplification to ensure the amplicon had a comparable melting temperature when compared to positive control. NRTCs and no template controls were included in all reactions. All standards, samples and controls were run in duplicate. All qPCR reaction efficiencies were >90% and all PCR products were run on gel electrophoresis to ensure correct amplicon size. Data were analysed using Relative Expression Software Tool (REST) (Pfaffl et al., 2002). *CbSITL* expression was normalized to the *EFL* and *RPS1* reference genes. We applied a randomization test within the REST software (2000 randomisations, Pair Wise Fixed Reallocation Randomisation Test) to determine statistically significant differences from the control.

### *C. braarudii SITL* expression in environmental populations

Samples were collected at L4 Station (4°13 W 50°15 N) in the Western English Channel. Both 15 and 50 μM plankton nets were towed behind RV *Sepia* for 10 min and the contents resuspended in 2 L of surface seawater. To positively identify *C. braarudii*, initial observation was conducted by light microscopy using a Leica DMi8 Inverted Microscope with a DFC7000 T colour camera (Leica Microsystems, UK). To confirm identity, 10 ml of the 15 μM net plankton sample was prepared for scanning electron microscopy (SEM). Samples for SEM were filtered onto a 13 mm 0.4 μm Isopore filter (Millipore EMD) and rinsed with 5 ml MilliQ water to remove any salt. Filters were air dried, mounted onto an aluminium stub and sputter coated with 10 nM Au/Pd (Emitech K550; Quorum Technologies, UK). The sample was analysed using a Jeol JSM‐6610LV SEM. For RNA preparation, 200 ml of sample from each net was centrifuged at 3800*g* at 4°C for 10 min. The RNA was extracted from the pellet, cDNA prepared and qPCR conducted as described above. qPCR reactions for were conducted for *C. braarudii SITL* and *EFL* genes. Positive qPCR amplicons were sequenced to confirm identity.

### Si transporters in eukaryote Ocean Gene Atlas metatranscriptome

Sequence similarity searches were conducted of the Ocean Gene Atlas metatranscriptome, MATOUv1 + T database (Marine Atlas of Tara Ocean Unigenes, https://tara-oceans.mio.osupytheas.fr/) (Villar et al., 2018) using *CbSITL* and *SaSIT1* as queries. Multiple sequence alignments of environmental SITs and SITLs were generated using MUSCLE and manually inspected for alignment quality. After manual refinement of the alignment, GBLOCKS 0.91b was used to remove poorly aligned residues and then ProtTest was used to determine the best substitution model (WAG with gamma and invariant) for phylogenetic analysis. Maximum likelihood phylogenetic trees were generated using MEGAX software with 100 bootstraps.

### Phytoplankton DSi drawdown assay and BSi incorporation

Media for Si‐drawdown experiments was prepared from Gulf Stream seawater (as described above) but with no added Si. The [DSi] was measured prior to adding sufficient Na_2_SiO_3_.5H_2_O to achieve 5 μM Si, a value that represents environmentally relevant [Si] and within the range of K_m_ values reported for diatom Si transporters (Curnow et al., 2012; Knight et al., 2016; Thamatrakoln & Hildebrand, 2008b). Cells previously acclimated to 5 μM Si for several generations were harvested from cultures at early exponential phase and gently washed in Si‐free media using a Nalgene polycarbonate filter unit. Replicate 200 ml cultures were seeded with washed cells for a starting density of 1–5 × 10^4^ ml^−1^ and maintained at the same temperature and light conditions as above. On each sampling day, cell counts, and aliquots for DSi, and BSi were taken. Fifteen mL culture aliquots were 0.2 μm filtered (Merck Millipore Ltd.) using lubricant free sterile syringes (to avoid Si contamination) and filtrate was stored at 4°C prior to DSi analysis. The filters with cells were frozen at −20°C for BSi analysis. Three no‐cells flasks were also included as a negative control in every experiment, and a diatom positive control was also included in the first sets of experiments (providing two replicates).

[DSi] was determined using a silicate molybdate‐ascorbate assay (Kirkwood, 1989) modified from Brzezinski and Nelson (1995) and Brzezinski et al. (1997). Measurements from the Si draw‐down experiment were conducted at UNCW Centre for Marine Science using an AutoAnalyzer3 (Bran Luebbe, Germany) with a range of calibration standards from 1.66 to 4.99 μM. Oxalic acid concentrations were increased to saturated levels (143 g/L) to overcome any phosphate interference. Molybdate was made fresh for each run.

For BSi determination, the previously collected and frozen drawdown assay filters were processed using the alkaline digestion method as described by Brzezinski and Nelson (1995), with modifications from Paasche (1980) and Krausse et al. (1983). For coccolithophores, filters were first treated with 1 ml 0.5 M HCl to fully dissolve coccoliths and subsequently neutralized using NaOH before alkaline digestion. For the alkaline digestion, each filter was placed in a 15 ml polymethylpentene tube (Diagenode, Inc.) with 4 ml of 0.2 M NaOH and brought to 100°C in a water bath for 20 min, cooled, and neutralized with 1 ml of 0.5 M HCl. The digest was centrifuged at 10,000 rpm for 9 min and aliquots of supernatant were removed and diluted for autoanalyser analysis as appropriate. Analytical blanks with filter only (no cells) were included for each run.

### Measurement of Si uptake using ^32^Si

*C. braarudii* was grown in f/2 media without additional Si at 16 ± 1°C under a 12:12 h light: dark cycle at ~120 μmol photons m^−2^ s^−1^ (EnviroGro T5 Hydrofarm). Exponentially growing cells were inoculated into triplicate cultures at a cell density of ~6000 cells ml^−1^ and an ambient Si concentration of 9.65 μM. Three treatments were tested: live cells in f/2 media, dead cells (heat treated 10 min at 60°C before the incubation), and Ge‐treated (live cells, but with the addition of 1.08 ml of 1 mM GeO_2_ to give a ratio of Ge:Si of 0.75). The cultures were then spiked with 7423 dpm of ^32^Si(OH)_4_ to start the incubation and cells were sampled at 4 and 24 h by filtration onto 0.6 μm polycarbonate filters. The filters were placed on planchettes, air dried, then covered with mylar. ^32^Si activity was evaluated after samples had reached secular equilibrium between ^32^Si and its daughter ^32^P using low‐level beta counting (Krause et al., 2011). The experiment was repeated using *E. huxleyi*, representing a control coccolithophore species that does not require Si. The detection limit on cell uptake was calculated by determining the variability in the triplicate measurements of radioactivity, adjusted for the amount of cold Si taken up per unit volume (2SD of DPM × Si/DPM), divided by the mean cell abundance.

### Energy dispersive x‐ray spectroscopy

A Zeiss Auriga SEM equipped with a Bruker Quantax X‐ray detector and analysis software was used for energy‐dispersive x‐ray spectroscopy (EDS). Spectra comprising 500 K x‐ray photon counts were collected from a minimum of three cells and five regions for each species. Column conditions were set to achieve 3500–5000 counts per second and the dead time typically ranged between 1% and 2%. The software allowed for x‐ray peak detection and standardless elemental quantification resulting in estimates for weight percent and atomic percent for the identified elements.

### Determination of Si/Ca in coccoliths

Coccolith samples were transferred to pre‐cleaned 2.0 ml microcentrifuge tubes and further rinsed by adding 1000 μl milli‐Q water to each sample, sonicating to mix the suspension and centrifuging at 4000 rpm for 8 min. The supernatant water was removed and the rinse procedure repeated twice more. After the third rinse and removal of the overlying water, the samples were dissolved by adding 650 μl of 0.1 M HNO_3_ to each. The solutions were centrifuged and 600 μl supernatant transferred to a clean tube and saved for analysis. A 25 μl aliquot was diluted 20 fold for Ca determination by ICP‐OES (de Villiers et al., 2002).

Samples for Si/Ca determination were diluted in 0.1 M HNO_3_ to give [Ca] of 200 ppm and Si/Ca ratios determined on a Thermo ElementXR sector field ICP‐MS at the Department of Earth Sciences, University of Cambridge. A platinum injector and platinum cones were used in order to minimize the Si background, together with a PFA cyclonic spraychamber and a 50 μl min^−1^ PFA nebulizer. The Si background signal was further reduced by operating the instrument at reduced plasma RF power of 1050 W, compared to the regular hot plasma of 1200 W.

Standards were prepared by adding Si to existing multi‐element standard solutions (Misra et al., 2014) to give a series of standards each containing 200 ppm Ca in 0.1 M HNO_3_ with Si concentrations increasing from 0 to 24 ppb, covering the range in Si/Ca to 171 μmol/mol. The cones were cleaned before running and conditioned at the start of the run by aspirating 100 ppm Ca solution in 0.1 M HNO_3._ Following initial tuning in low resolution, the instrument was switched to medium resolution (μm/m = 4000) in order to separate Si isotopes from interfering isobars and optimized for Si using a standard solution containing 16 ppb Si in 200 ppm Ca. The measurement sequence included ^23^Na, ^28^Si, ^29^Si and ^43^Ca with Si/Ca calculated from the measured ^28^Si/^43^Ca ratio. The minor ^29^Si isotope was used for verification and Na/Ca was measured to check the efficiency of the sample cleaning and rinsing procedures.

Cellular Si quota was calculated using the measured Si/Ca ratios (Table 1) and published cellular Ca quotas: for *E. huxleyi* 0.67 pmol Ca per coccosphere (Langer et al., 2009; Langer & Benner, 2009; Oviedo et al., 2014), for *C. braarudii* 35.89 pmol Ca per coccosphere (Sheward et al., 2017), for *C. leptoporus* 11 pmol Ca per coccosphere (Langer et al., 2006; Langer et al., 2012).

**TABLE 1:** Si content of coccoliths from three coccolithophore species, determined by mass spectrometry

Species	DSi in media (μM)	Requirement for Si?	^28^Si/Ca (μmol/mol)	SD	Si/Coccosphere (fmol)
*E. huxleyi*	2	No	7.92	±0.89	0.01
*C. leptoporus*	5	Yes	6.24	±1.72	0.07
*C. braarudii*	10	Yes	55.49	±1.16	1.99

## RESULTS

### CpSITL is a Na^+^‐coupled Si transporter

**FIGURE 1:**
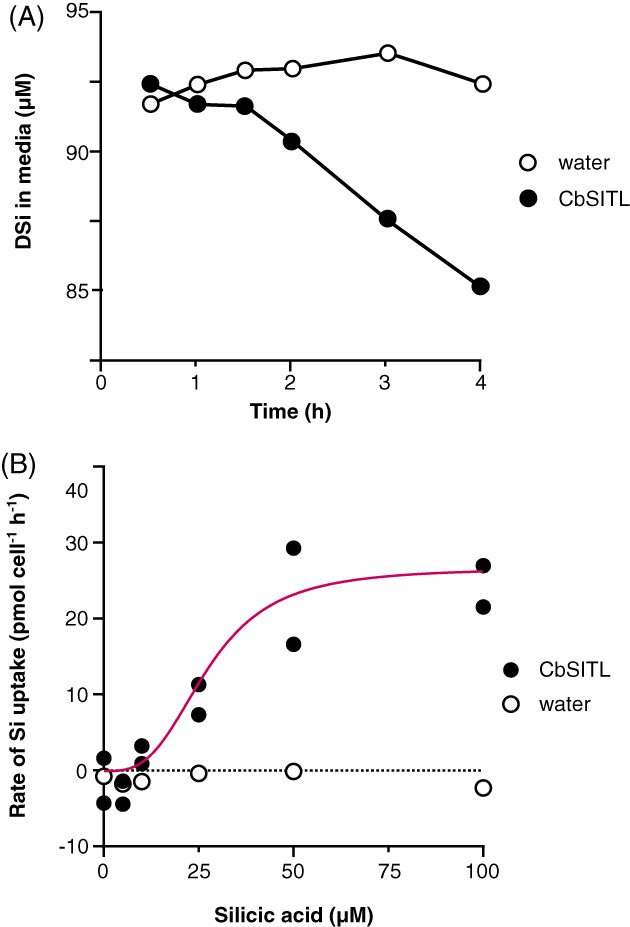
Heterologous expression of CbSITL in *Xenopus* oocytes. (A) Measurement of silicic acid drawdown from the media surrounding *Xenopus* oocytes injected with either water or CbSITL mRNA. The external concentration of silicic acid was 100 μM. A single representative experiment (*n* = 1) is shown to illustrate how rates of Si uptake were calculated for Figure 1B. Each sample represents 50 injected oocytes. (B) Transport of Si by CbSITL at different concentrations of external DSi. Expression of CbSITL leads to an apparent sigmoidal increase in Si transport with increasing DSi that is absent in water‐injected controls. The CbSITL data do not fit well to the Michaelis–Menten equation and instead is fit here to the Hill equation as shown. The same fitting procedure was attempted on all data but did not converge on a solution for the control samples. At each concentration, *n* = 2 for oocytes injected with *CbSITL* and *n* = 1 for oocytes injected with water, with 50 oocytes injected per sample.

We first determined whether the novel SITL proteins found in coccolithophores could act as Si transporters when expressed in heterologous systems. *Xenopus* oocytes were microinjected with mRNA coding for *C. braarudii* SITL (CbSITL). Western blots targeting the V5‐epitope tag indicated a strong band at approximately 45 kDa, close to the predicted MW of CbSITL monomer, with a weaker band present at 90 kDa that may be indicative of dimerization (Figure S1). Oocytes expressing CbSITL were able to substantially deplete DSi from the external media, whereas no decrease in DSi was observed in control oocytes injected with water (Figure 1A,B). Calculation of uptake kinetics indicated that CbSITL expressed in *Xenopus* oocytes has a similar affinity for silicic acid (*K*_*m*_ = 27.4 ± 4.0 μM) to diatom SITs expressed in *Xenopus* (*Cylindrotheca fusiformis* SIT1, *K*_*m*_ 30 μM; Hildebrand et al., 1997).

**FIGURE 2:**
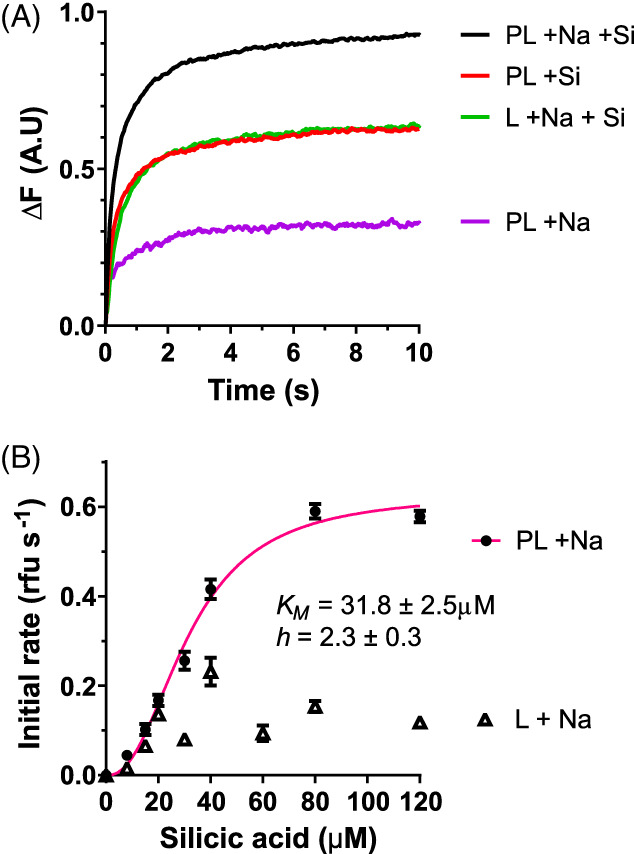
CbSITL exhibits Na^+^‐dependent Si transport activity in yeast proteoliposomes. (A) Individual representative traces indicating change in apparent fluorescence emission when reconstituted yeast proteoliposomes containing CbSITL are exposed to 80 μM silicic acid. PL, proteoliposomes (containing CbSITL); L, liposomes (no CbSITL); +Si, silicic acid present; +Na, 150 mM external sodium present. The increase in apparent fluorescence is due to formation of Zn silicate salts within the proteoliposome. Note that incorporation of CbSITL increases the Si transport beyond that of liposomes (without CbSITL) or proteoliposomes in the absence of Na^+^. (B) Initial (linear) rates versus silicic acid concentration. Proteoliposomes show a sigmoidal function while liposomes essentially show no effect. Experiments are mean ± s.d. (*n* = 3) from three independent batches of (proteo)liposomes prepared from two independent protein preparations. The same fitting procedure was attempted on all data, but it was not possible to converge on a solution for the control samples.

We next characterized the Si transport activity of CbSITL in reconstituted yeast proteoliposomes (Knight et al., 2016). Recombinant CbSITL expressed in the membrane fraction of *S. cerevisiae* cells was purified by affinity chromatography. Western blotting using the V5‐epitope tag indicated bands corresponding to the approximate size of CbSITL monomers and dimers, as well as higher monomeric forms that may represent a degree of aggregation (Figure S2). Si transport activity into the proteoliposomes was measured by the change in apparent fluorescence signal due to the formation of zinc silicates (Knight et al., 2016). In the presence of Na^+^ and Si in the external medium, proteoliposomes containing CbSITL demonstrated Si transport activity (Figure 2). In the absence of Na^+^, no Si transport was observed above the level of non‐specific Si transport shown by control liposomes that lack CbSITL. This indicates that CbSITL is a Na^+^‐dependent Si transporter, as is the case for diatom SITs (Hildebrand et al., 1997; Knight et al., 2016). The affinity of CbSITL for silicic acid estimated from the proteoliposome assay (*K*_*m*_ 31.8 ± 2.5 μM) was very similar to that estimated from *Xenopus* oocytes. The *K*_*m*_ of diatom SITs estimated by the proteoliposome assay was 19.1 and 19.6 μM for PtSIT1 and NaSIT1 (Knight et al., 2016). In combination, our results demonstrate that CbSITL exhibits many similar characteristics to diatom SITs and indicate that coccolithophores possess mechanisms for active transport of DSi. More broadly, the results provide experimental evidence that the SITLs represent a novel family of Si transporters that are widely distributed amongst eukaryotes (Marron et al., 2016).

### CbSITL is transcriptionally regulated by Si availability

**FIGURE 3:**
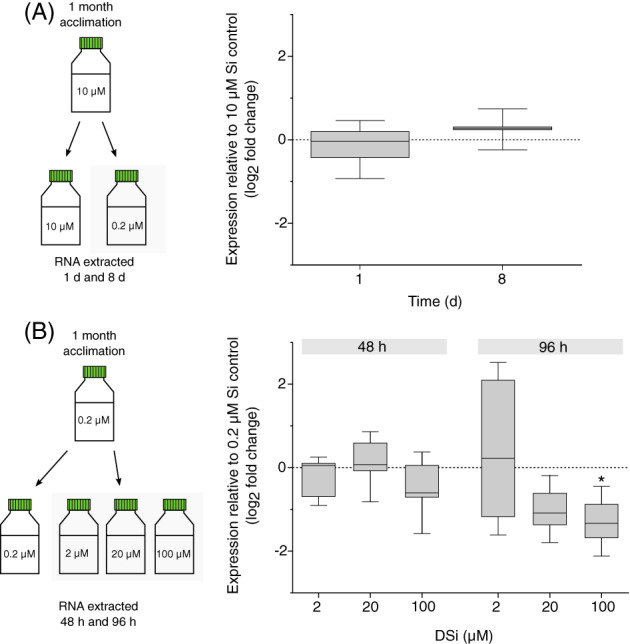
Transcriptional regulation of *CbSITL* by Si. (A) *CbSITL* expression in response to low Si. *C. braarudii* cultures were grown in 10 μM Si for 1 month then transferred to control (10 μM) and very low (0.22 μM) Si conditions (*n* = 3). Gene expression of *CbSITL* was analysed by RT‐qPCR after 1 and 8 days of incubation. No significant difference in *CbSITL* expression in low Si relative to control was observed over 8 days of incubation (Pair Wise Fixed Reallocation Randomisation Test, REST software). Box plots show the interquartile range of the permuted expression values calculated by the randomisation test, whiskers represent minimum and maximum values. The line represents the median value. (B) Expression of *CbSITL* in response to Si replenishment. *C. braarudii* cultures were grown in 0.22 μM Si for 1 month then transferred to 0.22 (control), 2, 20 and 100 μM Si (*n* = 3). *CbSITL* expression was analysed by RT‐qPCR relative to the low Si control after 48 and 96 h. No significant difference in the expression of *CbSITL* was observed at 2, 20 and 100 μM Si after 48 h of incubation, but expression of *CbSITL* was significantly down regulated in the 100 μM Si treatment after 96 h. (* denotes *p* < 0.05, Pair Wise Fixed Reallocation Randomisation Test, REST software). Box plots show the interquartile range of the permuted expression values calculated by the randomization test, whiskers represent minimum and maximum values. The line represents the median value.

SITs in diatoms and choanoflagellates are transcriptionally regulated by the availability of DSi, exhibiting strong upregulation in Si‐limited cultures (Marron et al., 2016; Thamatrakoln & Hildebrand, 2007). We therefore examined the expression of *CbSITL* in response to changes in DSi in the seawater media using RT‐qPCR. We first examined the effect of Si limitation by transferring *C. braarudii* cells grown at 10 μM DSi to low DSi (0.22 μM). We found that *CbSITL* exhibited no significant change in expression after 1 day or 8 days, relative to cells maintained at 10 μM DSi (Figure 3A). However, whilst highly silicified diatoms and choanoflagellates would likely become limited for Si within this time period, limitation of *C. braarudii* growth only occurs after extended period in low Si (>18 days) (Walker et al., 2018). To counter the possibility that cellular Si pools had not been fully depleted, we examined whether CpSITL expression was repressed when cells were transferred from low to high DSi. *C. braarudii* was grown at low DSi (0.22 μM) for 28 days and then transferred to a range of higher DSi concentrations (up to 100 μM). Although no significant effects were observed after 48 h, cells transferred to the highest DSi concentration (100 μM) exhibited a significant decrease in *CbSITL* expression after 96 h (Figure 3B). We conclude that *CbSITL* can be transcriptionally regulated by DSi availability, in a manner similar to SITs in highly silicified organisms (Brembu et al., 2017; Marron et al., 2016) although the time‐scale for response to changing Si appears to be much longer in coccolithophores.

### Environmental expression of Si transporters in coccolithophores

We next examined whether Si transporters were expressed in natural populations of coccolithophores. *C. braarudii* is commonly found in the western English Channel in late summer/early autumn, where nutrient concentrations, including Si, are typically low due to seasonal stratification and high phytoplankton productivity throughout summer (Smyth et al., 2010). The presence of *C. braarudii* in samples of surface seawater was monitored by light microscopy and confirmed by SEM ( Figure S3). We then extracted RNA and used RT‐qPCR to examine gene expression. We detected transcripts of a reference gene for *C. braarudii* (*EFL*), alongside transcripts for *CbSITL*, indicating that *CbSITL* is expressed by environmental *C. braarudii* populations (Figure S3). The concentration of DSi in surface water at the time of sampling was 0.66 μM.

To further probe the requirement for active Si transport in coccolithophore communities, we examined the Tara Oceans eukaryote metatranscriptomics dataset (Villar et al., 2018) for evidence of global expression of coccolithophore SITs and SITLs. We found several novel SIT and SITL sequences that exhibited a high similarity to those previously identified in coccolithophores (Durak et al., 2016) ( Figure S4). The novel SITL sequences formed a well‐supported clade with other haptophytes that was distinct from SITL clades including metazoa or foraminifera. As SITL sequences have not been found in any non‐calcified haptophyte species, we propose that the environmental haptophyte SITL sequences likely belong to coccolithophores. The environmental SIT sequences formed a well‐supported clade with SITs from the coccolithophore *S. apsteinii* and the silicifying haptophyte *P. neolepis*. Within this clade, several SIT sequences exhibit very high similarity to *P. neolepis*, although the identity of the other environmental haptophyte SITs is not clear. The coccolithophore SIT and SITL sequences were recovered from a broad geographic distribution, encompassing the Pacific, Atlantic and Indian Oceans, and were primarily found in samples with low Si concentrations (Figure S5).

### Measuring Si uptake rates in coccolithophores

**FIGURE 4:**
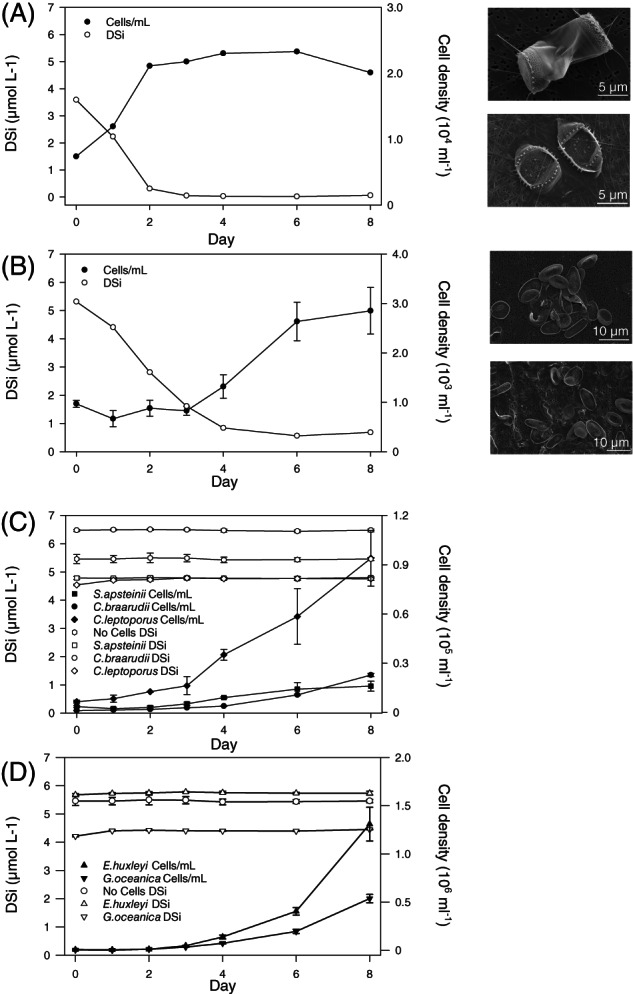
Si drawdown by biomineralized phytoplankton. (A) The cell density and concentration of dissolved Si (DSi) were measured over a period of 8 days in a culture of the diatom *Thalassiosira weissflogii*. SEM micrographs show silicified structures from *T. weissflogii* Day 0 (top) and Day 8 (bottom). (B) Growth and DSi concentration in a culture of the silicifying haptophyte *Prymnesium neolepis*. SEM micrographs show silicified scales on Day 0 (top) and Day 8 (bottom). (C) Si drawdown by coccolithophore species that possess Si transporters and are sensitive to Ge (*Scyphosphaera apsteinii*, *Coccolithus braarudii* and *Calcidiscus leptoporus*). (D) Si drawdown by coccolithophore species that do not possess Si transporters and are insensitive to Ge (*Emiliania huxleyi* and *Gephyrocapsa oceanica*). The silicifying organisms exhibit a rapid drawdown of DSi to almost 0 μM, whereas no significant decrease in DSi was observed in any coccolithophore culture. The initial concentration of DSi was 5 μM. *n* = 4 except for *T. weissflogii* where *n* = 2, standard error bars are indicated.

We next examined evidence for Si uptake by *C. braarudii* and a range of other coccolithophore species. In batch culture, heavily silicified organisms can rapidly deplete DSi from the surrounding media, providing a simple assay to examine rates of DSi uptake. The diatom *Thalassiosira weissflogii* depleted DSi from the media within 3 days (initial concentration 4 μM) (Figure 4A). The silicifying haptophyte *Prymnesium neolepis* also rapidly depleted DSi in a similar manner (initial concentration 5 μM DSi), (Figure 4B). Interestingly, *P. neolepis* was able to grow after DSi was extensively depleted, although cells possessed fewer scales or were naked. In contrast to the silicifying organisms, we could not detect any drawdown of DSi by the coccolithophores *S. apsteinii*, *C. braarudii*, and *C. leptoporus* when monitored over an 8 days period (Figure 4C). These three species all possess Si transporters and are sensitive to Ge (Durak et al., 2016; Walker et al., 2018). The Ge‐insensitive coccolithophores *E. huxleyi* and *G. oceanica*, which lack Si transporters and have no known physiological Si requirement, also showed no detectable drawdown of DSi in this assay (Figure 4D). Si depletion strongly disrupted silica formation in *T. weissflogii* and *P. neolepis*, whereas the coccolithophores did not exhibit any change in coccolith morphology throughout the experiment (Figure 4; Figure S6). The Si requirement of coccolithophores is therefore likely to be much lower than for highly silicified organisms, in which Si forms a key structural component of their biomineralized cell coverings.

Given the very low expected rates of Si transport inferred by the Si depletion assays, we used uptake of radiolabelled ^32^Si(OH)_4_ as a more sensitive measure of Si transport. Uptake of ^32^Si has been used extensively to determine the rate of Si uptake in diatoms and other silicifying organisms (Krause et al., 2011; McNair et al., 2018). The uptake of radioactivity by live cells was compared to dead cells (heat‐treated) to quantify passive absorption/adsorption of Si and we also examined whether Ge was able to inhibit uptake (0.75 Ge/Si ratio). The radioactivity taken up by live cells did not exceed that of dead cells in either *C. braarudii* or *E. huxleyi* after 4 h or 24 h (t‐test, *p* = 0.05, d.f. = 2), indicating that there was no detectable active uptake of Si (Tables S2 and S3). Ge also had no measurable effect on Si uptake (t‐test, *p* = 0.05, d.f. = 2). Therefore, any Si uptake by *C. braarudii* cells under these conditions is occurring at a rate lower than the limit of detection. *T. weissflogii* typically shows uptake rates of 0.5–1 pmol Si cell^−1^ d^−1^ (De La Rocha et al., 2010). Based on the variability of the measurements, we calculated that the detection limit of the ^32^Si assay for *C. braarudii* was approximately 0.006 ± 0.003 pmol Si cell^−1^ d^−1^. Si uptake rates in *C. braarudii* must therefore be at least 100‐fold less than that of *T. weissflogii*, a diatom of similar cell size.

To put these findings in context of cellular Si quotas, we next measured Si incorporation into biogenic structures (biogenic Si, BSi). We found that *T. weissflogii* and *P. neolepis* contain 5.99 and 9.09 pmol BSi cell^−1^, respectively. The high BSi content of *P. neolepis* likely reflects its prolific production of silicified scales, arranged in multiple layers around the cell (Yoshida et al., 2006). In contrast, BSi was not significantly different from background in five coccolithophore species (one‐way ANOVA, *p* > 0.05) (Table S4). As the BSi analysis measures both amorphous and soluble Si pools, these results indicate that coccolithophores do not accumulate unmineralized Si, such as observed in some cyanobacteria (Baines et al., 2012).

### Si incorporation into coccoliths

**FIGURE 5:**
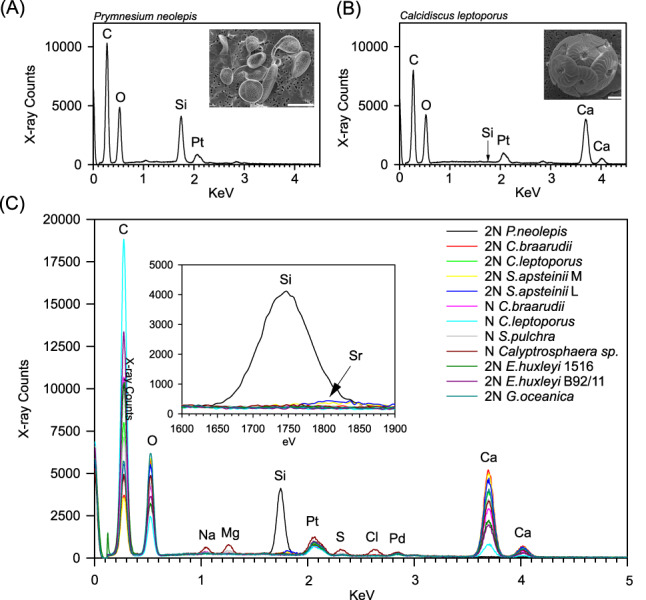
Elemental analysis of Si in coccoliths. (A) Elemental analysis of a silica scale from the silicifying haptophyte (*Prymnesium neolepis*) using energy‐dispersive x‐ray spectroscopy (EDS). (B) EDS of a coccolith from *Calcidiscus leptoporus*. Insets show SEM micrographs of each species. Scale bars are 10 μm for *P. neolepis* and 5 μm for *C. leptoporus*. For *C. leptoporus*, the arrow represents where the energy reading for Si would be. (C) A comprehensive EDS analysis across biomineralized haptophytes including *P. neolepis* and a combination of haploid (N) and diploid (2N) coccolithophore species. Inset shows Si peak for *P. neolepis* and the absence of Si in all coccolithophore species. A small Sr peak was observed for *Scyphosphaera apsteinii* (arrow).

Whilst the total cellular Si content of coccolithophores appears to be very low, Si limitation (and/or disruption of Si uptake using Ge) has specific impacts on coccolith morphology (Durak et al., 2016; Langer et al., 2021; Walker et al., 2018). We therefore examined whether we could detect and quantify Si in coccoliths. Previous measurement using EDS has suggested that the large barrel‐shaped lopadoliths of *S. apsteinii* may contain small amounts of Si (Drescher et al., 2012). However, measurements of low levels of Si using EDS can be problematic due the potential for contaminating signals from substances used in sample preparation. We therefore extensively modified our protocols to ensure that low levels of Si could be measured reliably and accurately using this approach (see Experimental Procedures). Using this improved methodology, we were unable to detect substantial amounts of Si in coccoliths from any coccolithophore, and there was no significant difference in Si concentration between species that require Si and those that do not (Figure 5, Table S5).

We next examined the Si content of coccoliths directly using a mass spectroscopy approach to allow greater sensitivity. Again, rigorous methodological adaptations were necessary to prevent contamination of the samples with Si during cleaning and preparation for analysis (see Experimental Procedures). These analyses confirmed that the Si content of coccoliths is indeed very low with 55.49 ± 1.16 μmol ^28^Si per mol of Ca in *C. braarudii*. Although this value was substantially greater than that observed in *E. huxleyi*, the Si content of another Si‐requiring species *C. leptoporus* was not significantly different from *E. huxleyi*. The ratio of Si to Ca allowed us to calculate cellular quotas for Si associated with coccoliths (Table 1). These low levels indicate that Si is unlikely to be a structural component of coccoliths. Instead, we hypothesize that Si is required during the process of coccolith formation, acting to support the correct morphogenesis of the growing coccolith crystals (Langer et al., 2021), rather than becoming incorporated into the coccolith itself. In other terms, Si is acting as a micronutrient in coccolithophores, whereas it is a macronutrient in silicifying organisms.

## DISCUSSION

Although silicon is the second most abundant element in the Earth's crust, our understanding of its role in biological systems remains limited and relatively few proteins have been identified that play a direct role in Si biology. In terms of Si transport, it is now clear that aquaporins can play an important role in facilitating the diffusion of Si(OH)_4_ across biological membranes (Coskun et al., 2021). Other than the 10‐TM SITs, active transport of Si has been demonstrated by Lsi2, a protein related to ArsB arsenate transporters that plays an important role in Si transport in plants, and SLC34a2, which has a proposed role in Si transport in vertebrates (Ma et al., 2007; Maldonado et al., 2020; Ratcliffe et al., 2017). However, both Lsi2 and SLC34a2 function in Si efflux from cells and are therefore unlikely to mediate Si uptake from the environment directly. The SITs are therefore the only previously characterized eukaryote Si transporters that have been shown to facilitate energized uptake of Si across the plasma membrane (Hildebrand et al., 1997; Knight et al., 2016). Our results show that the SITLs represent an additional class of eukaryote Si transporters that can facilitate cellular uptake of Si and actively deplete DSi from the external media when expressed in heterologous systems. SITLs are found in wide range of marine eukaryotes in addition to coccolithophores, including foraminifera, dinoflagellates, radiolarians, dictyochophytes and copepods, suggesting that the capacity for active Si uptake may be widespread amongst these major plankton lineages (Durak et al., 2016; Marron et al., 2016).

Many secondary active transporters exhibit an inverted repeat topology and likely evolved via duplication and fusion of an ancestral protein that originally acted as a homodimer (Keller et al., 2014). Incorporating both domains in a single protein likely allowed functional specialization of each transporter, although there are relatively few examples of secondary active transporters where the ancestral precursors have been clearly identified and characterized in order to compare their biochemical properties (one example is the SWEET and semi‐SWEET sugar transporters) (Feng & Frommer, 2015; Keller et al., 2014). The widespread presence of the SITs and SITLs in eukaryotes, along with the likelihood that SITs have evolved from SITLs on multiple independent occasions (Marron et al., 2016), provides an excellent system through which the evolution of secondary active transporters can be studied. Our initial characterization of the biochemical properties of SITLs demonstrates that they have a similar affinity for Si to diatom SITs. Moreover, computational analysis predicts that the structure of a SITL homodimer is remarkably similar to diatom SITs (Knight et al., 2022). However, there are clear delineations in the taxonomic distribution of these transporters, with SITs found primarily in heavily silicified lineages (diatoms, choanoflagellates, chrysophytes, synurophytes and *P. neolepis*) and SITLs found in lineages that are either non‐silicified or partially silicified (Marron et al., 2016). This distribution points to a functional advantage of SITs over SITLs in organisms where a high cellular rate of Si transport is required.

A single coccolithophore species (*S. apsteinii*) has been found to possess both a SIT and SITL (Durak et al., 2016). We did not find evidence for a larger BSi content in *S. apsteinii* or increased rates of DSi uptake in this species compared to other coccolithophores. The reason for the additional presence of a SIT in *S. apsteinii* are therefore unclear given that SITs are primarily associated with heavily silicified lineages. The identification of further SITs related to *S. apsteinii SIT1* in the Tara Oceans metatranscriptome could be indicative of the wider presence of SITs in other coccolithophore species. However, these sequences may also represent SITs from other uncharacterized haptophytes. A potential candidate is *Petasaria*, a silicified genus of flagellated protists that has been tentatively assigned to the haptophytes, although its taxonomic position remains uncertain and it has yet to be characterized at the molecular level (Jordan et al., 2015; Moestrup, 1979; Patil et al., 2015).

We have demonstrated that the SITLs found in coccolithophores are active as Si transporters, but we have also shown that the cellular quota for Si in all coccolithophores is very low, bringing into question the requirement for specific Si transport across the plasma membrane. Active Si transport in coccolithophores may act primarily to enable acquisition of Si from seawater where DSi is very low, rather than to support high‐capacity transport. In other words, it is the affinity of the transporters rather than their capacity that may be the defining characteristic of their function (Flynn et al., 2018). At higher concentrations of DSi (>30 μM), diatoms acquire Si primarily by diffusion of non‐dissociated silicic acid, while active Si transport by SITs is the primary mechanism of Si uptake at lower concentrations of DSi (Thamatrakoln & Hildebrand, 2008a). As DSi concentrations in much of the surface ocean can be <5 μM, diatom SITS and CbSITL do not show a particularly high affinity for Si when expressed in heterologous systems (K_m_ 20–30 μM). However, diatoms themselves exhibit higher affinity Si uptake (Thamatrakoln & Hildebrand, 2008), suggesting that SITs may exhibit different characteristics in vivo.

Diatom SITs show strong upregulation within hours of Si starvation and conversely exhibit rapid downregulation when Si‐limited diatoms are shifted to high DSi (Brembu et al., 2017; Shrestha et al., 2012; Smith et al., 2016). SITs from silicifying choanoflagellates also show a similar pattern of transcriptional regulation, although the fold changes in gene expression were more modest than observed in diatoms and were only measured after 48 h (Marron et al., 2016). In *C. braarudii*, repression of *CbSITL* following Si addition to Si‐limited cells only occurred after 96 h and upregulation of *CbSITL* was not observed even after Si limitation for 8 days. These differences may be due in part to the very different rates of Si utilization between coccolithophores and silicified organisms. Exponentially growing diatoms only have a small intracellular pool of Si and can rapidly deplete DSi from the external medium, leading to Si starvation within hours following a shift to low DSi (Brembu et al., 2017; Thamatrakoln & Hildebrand, 2008). However, more recent work using EDS to measure cellular Si concentrations in cryo‐fixed cells (rather than the release of labile Si by boiling) suggested that diatoms actually maintain a much larger Si pool (with concentrations around 150 mM), which increases during Si starvation (Kumar et al., 2020). These very different conclusions may be due to the differing abilities of these approaches to detect Si in the form that it is stored within the cell (Kumar et al., 2020). Although the intracellular Si pool of coccolithophores was also very low (when measured by release of labile Si), coccolithophores do not substantially deplete DSi from the external medium and Si limitation was only observed after prolonged growth at very low DSi (<0.2 μM), suggesting very slow turnover of the intracellular Si pool (Langer et al., 2021; Walker et al., 2018). It is therefore possible that intracellular Si was not depleted after 8 days at low DSi (0.22 μM) in our qRT‐PCR experiments. However, a low rate of Si utilization does not explain the delayed repression of *CbSITL* expression following a shift to high DSi, as we would expect the very small intracellular Si pools to be rapidly replenished. Addition of high concentrations of DSi to Si‐limited diatoms results in a very large increase in the cellular Si pool within minutes (Thamatrakoln & Hildebrand, 2008). One possible explanation is that non‐silicifying organisms such as coccolithophores have a much lower permeability to silicic acid than diatoms, as uncontrolled diffusive entry of Si would be undesirable in the absence of a large intracellular sink for Si. In this scenario, active uptake of Si through SITLs over a wide range of external DSi would allow much tighter control of the rates of Si uptake than the combination of active transport and diffusive entry found in diatoms.

SITs play a direct role in Na^+^‐coupled Si uptake across the plasma membrane in diatoms. As the SITLs are also Na^+^‐dependent transporters, it seems likely that their primary role in other organisms will also be to facilitate Si transport across the plasma membrane, utilizing the large inward Na^+^ gradient. However, as direct evidence for Si uptake across the plasma membrane of coccolithophores remains lacking, we must consider the possibility that coccolithophore SITLs perform alternative cellular roles. The localization of SITLs has not been determined, so we cannot rule out the possibility of localization to endomembranes, such as the coccolith vesicle. In this scenario, the cell could rely on diffusive uptake of Si across the plasma membrane in most environments to support its low cellular Si quota (assuming it can maintain a sufficiently low DSi concentration in the cytoplasm to create an inward diffusive gradient), with SITLs acting to regulate Si concentration within the coccolith vesicle during calcification. It should also be noted that alternative roles for SITs have been proposed in diatoms. TpSIT3 from *Thalassiosira pseudonana* does not localize to the plasma membrane and is not transcriptionally regulated by DSi availability, in contrast to TpSIT1 or TpSIT2 (Shrestha & Hildebrand, 2015). It was proposed that TpSIT3 may therefore act in a regulatory role or as a cellular sensor of Si (Shrestha & Hildebrand, 2015). In the absence of measurable rates of Si uptake, it is possible that coccolithophore SITLs perform a role in Si sensing rather than Si transport. A number of membrane proteins that play dual role in transport and signalling (known as transceptors) have been identified in animals, fungi and plants, including NRT1.1, which acts as dual‐affinity nitrate transporter in *Arabidopsis*, but also mediates downstream responses to nitrate (Ho et al., 2009). Determining the localization of SITLs in coccolithophores and other organisms will be a critical key step in identifying their cellular role.

Calcification in coccolithophores evolved in the early Mesozoic around 220 Myr ago (Liu et al., 2010), at a time when the concentrations of DSi in the surface ocean were several orders of magnitude higher than at present, likely near 1 mM (Harper & Knoll, 1975; Hendry et al., 2018). The requirement for high‐affinity Si transport in ancestral coccolithophores is therefore uncertain as Si was unlikely to be limiting. However, the dramatic changes in Si biogeochemistry of the oceans have likely caused significant changes in the physiology of Si transport in marine organisms. The broad taxonomic distribution of the SITs, SITLs, and other Si transporters, such as Lsi2, strongly suggest that they have an ancient evolutionary origin (Marron et al., 2016). This points to a sophisticated capacity for Si transport in ancestral eukaryotes that may have been required to control cellular Si concentrations and limit the potential for toxic auto‐polymerization of silica in the high Si ocean. As Si concentrations in the surface ocean declined in the past 100 Myr, due primarily to biogenic control by diatoms, selective pressure to retain Si transporters for Si detoxification will have decreased, which may have contributed to their loss in multiple lineages. Organisms where Si played a physiological role will have encountered selective pressure to lower or lose their Si requirement and/or to enhance their capacity for Si acquisition (Hendry et al., 2018). Evidence for multiple losses of silicification is present in several lineages, such as sponges and centrohelids (Hendry et al., 2018; Maldonado, 2009; Zlatogursky, 2016). Coccolithophores may also have lowered their requirement for Si or lost it altogether, as appears to be the case in the Pleurochrysidaceae and the Noelaerhabdaceae (Durak et al., 2016; Langer et al., 2021). Selective pressure for mechanisms of higher affinity Si uptake as DSi concentrations in the surface ocean became lower has been proposed to have driven diversification of the SITs within the diatoms and the choanoflagellates (Hendry et al., 2018). It is likely that similar selective pressures may have influenced coccolithophore SITLs, leading to significant modification of their biochemical properties from those found in ancestral coccolithophores, which operated in a much higher DSi environment.

Our results show that coccolithophores possess the capacity for active Si transport, through the expression of SITLs, although we could not detect Si uptake across the plasma membrane. It remains to be determined whether SITLs function directly in Si uptake or play an alternative role within the cell. Whilst Si is essential for calcification in certain coccolithophore lineages, their cellular quota is very low. This means coccolithophores are much less likely to be susceptible to Si limitation than heavily silicified phytoplankton lineages and they will not contribute to significant Si drawdown themselves. However, under specific conditions where extensive drawdown of DSi by silicifiers occurs, such as following a large diatom bloom in seasonally stratified waters, DSi may reach submicromolar concentrations comparable to those that induce Si limitation of coccolithophores in laboratory studies (Langer et al., 2021; Walker et al., 2018; Yool & Tyrrell, 2003). Some natural coccolithophore populations may therefore experience Si limitation, but only in regions where Si is extensively depleted by the action of silicifying organisms. Coccolithophore populations in these regions may therefore have experienced selective pressure to lose their Si requirement in order to exploit this niche, but in other areas of the ocean that do not experience such extremes of Si depletion, we propose that Si availability is unlikely to strongly influence coccolithophore populations.

## AUTHOR CONTRIBUTION

Sarah Ratcliffe: investigation (equal lead). Erin M. Meyer: investigation (equal lead). Charlotte E. Walker: investigation (equal lead). Michael Knight: investigation(supporting). Heather M. McNair: investigation (supporting). Paul G. Matson: investigation(supporting). Debora Iglesias‐Rodriguez: investigation(supporting). Mark Brzezinski: investigation (supporting), review & editing (equal). Gerald Langer: investigation(supporting). Aleksey Sadekov: investigation (supporting). Mervyn Greaves: investigation(supporting). Colin Brownlee: review & editing (equal). Paul Curnow: conceptualization(equal), review & editing(equal). Alison R. Taylor: conceptualization (equal), investigation(supporting), review & editing (equal). Glen L. Wheeler: conceptualization(lead), investigation(supporting), original draft preparation (lead), review & editing(lead).

## CONFLICT OF INTEREST

The authors declare no competing interests.

## Supporting information


**Appendix S1.** Supporting Information.

## Data Availability

The data that support the findings of this study are available from the corresponding author upon reasonable request.

## References

[emi16280-bib-0001] Baines, S.B. , Twining, B.S. , Brzezinski, M.A. , Krause, J. , Vogt, S. , Assael, D. et al. (2012) Significant silicon accumulation by marine picocyanobacteria. Nature Geoscience, 5(12), 886–891.

[emi16280-bib-0002] Brembu, T. , Chauton, M.S. , Winge, P. , Bones, A.M. & Vadstein, O. (2017) Dynamic responses to silicon in *Thalasiossira pseudonana* ‐ identification, characterisation and classification of signature genes and their corresponding protein motifs. Scientific Reports, 7(1), 4865. 28687794 10.1038/s41598-017-04921-0 PMC5501833

[emi16280-bib-0003] Brzezinski, M.A. , Alldredge, A.L. & O'Bryan, L.M. (1997) Silica cycling within marine snow. Limnology and Oceanography, 42(8), 1706–1713.

[emi16280-bib-0004] Brzezinski, M.A. , Krause, J.W. , Baines, S.B. , Collier, J.L. , Ohnemus, D.C. & Twining, B.S. (2017) Patterns and regulation of silicon accumulation in *Synechococcus* Spp. Journal of Phycology, 53(4), 746–761. 28457002 10.1111/jpy.12545

[emi16280-bib-0005] Brzezinski, M.A. & Nelson, D.M. (1995) The annual silica cycle IN the Sargasso sea near Bermuda. Deep‐Sea Research Part I‐Oceanographic Research Papers, 42(7), 1215–1237.

[emi16280-bib-0006] Coskun, D. , Deshmukh, R. , Shivaraj, S.M. , Isenring, P. & Belanger, R.R. (2021) Lsi2: a black box in plant silicon transport. Plant and Soil, 466(1–2), 1–20. 34720209 10.1007/s11104-021-05061-1 PMC8550040

[emi16280-bib-0007] Curnow, P. , Senior, L. , Knight, M.J. , Thamatrakoln, K. , Hildebrand, M. & Booth, P.J. (2012) Expression, purification, and reconstitution of a diatom silicon transporter. Biochemistry, 51(18), 3776–3785. 22530967 10.1021/bi3000484

[emi16280-bib-0008] De La Rocha, C.L. , Terbruggen, A. , Volker, C. & Hohn, S. (2010) Response to and recovery from nitrogen and silicon starvation in *Thalassiosira weissflogii*: growth rates, nutrient uptake and C, Si and N content per cell. Marine Ecology Progress Series, 412, 57–68.

[emi16280-bib-0009] de Villiers, S. , Greaves, M. & Elderfield, H. (2002) An intensity ratio calibration method for the accurate determination of Mg/Ca and Sr/Ca of marine carbonates by ICP‐AES. Geochemistry Geophysics Geosystems, 3, 2001GC000169.

[emi16280-bib-0010] Drescher, B. , Dillaman, R.M. & Taylor, A.R. (2012) Coccolithogenesis in *Scyphosphaera apsteinii* (Prymnesiophyceae). Journal of Phycology, 48(6), 1343–1361. 27009987 10.1111/j.1529-8817.2012.01227.x

[emi16280-bib-0011] Durak, G.M. , Taylor, A.R. , Walker, C.E. , Probert, I. , de Vargas, C. , Audic, S. et al. (2016) A role for diatom‐like silicon transporters in calcifying coccolithophores. Nature Communications, 7, 10543. 10.1038/ncomms10543 PMC4742977 26842659

[emi16280-bib-0012] Durkin, C.A. , Koester, J.A. , Bender, S.J. & Armbrust, E.V. (2016) The evolution of silicon transporters in diatoms. Journal of Phycology, 52(5), 716–731. 27335204 10.1111/jpy.12441 PMC5129515

[emi16280-bib-0013] Feng, L. & Frommer, W.B. (2015) Structure and function of semisweet and sweet sugar transporters. Trends in Biochemical Sciences, 40(8), 480–486. 26071195 10.1016/j.tibs.2015.05.005

[emi16280-bib-0014] Flynn, K.J. , Skibinski, D.O.F. & Lindemann, C. (2018) Effects of growth rate, cell size, motion, and elemental stoichiometry on nutrient transport kinetics. PLoS Computational Biology, 14(4), e1006118. 29702650 10.1371/journal.pcbi.1006118 PMC5942848

[emi16280-bib-0015] Fowler, N. , Tomas, C. , Baden, D. , Campbell, L. & Bourdelais, A. (2015) Chemical analysis of *Karenia papilionacea* . Toxicon, 101, 85–91. 25981346 10.1016/j.toxicon.2015.05.007

[emi16280-bib-0069] Guillard, R.R.L. (1975) Culture of phytoplankton for feeding marine invertebrates in “Culture of Marine Invertebrate Animals.” In: Smith, W.L. & Chanley, M.H. (Eds.) New York: Plenum Press, pp. 26–60.

[emi16280-bib-0016] Harper, H.E. & Knoll, A.H. (1975) Silica, diatoms, and cenozoic radiolarian evolution. Geology, 3(4), 175–177.

[emi16280-bib-0017] Hendry, K.R. , Marron, A.O. , Vincent, F. , Conley, D.J. , Gehlen, M. , Ibarbalz, F.M. et al. (2018) Competition between silicifiers and non‐silicifiers in the past and present ocean and its evolutionary impacts. Frontiers in Marine Science, 5, 22.

[emi16280-bib-0018] Hildebrand, M. , Volcani, B.E. , Gassmann, W. & Schroeder, J.I. (1997) A gene family of silicon transporters. Nature, 385(6618), 688–689. 9034185 10.1038/385688b0

[emi16280-bib-0019] Ho, C.H. , Lin, S.H. , Hu, H.C. & Tsay, Y.F. (2009) CHL1 functions as a nitrate sensor in plants. Cell, 138(6), 1184–1194. 19766570 10.1016/j.cell.2009.07.004

[emi16280-bib-0020] Jordan, R.W. , Abe, K. , Cruz, J. , Eriksen, R. , Guerreiro, C. , Hagino, K. et al. (2015) Observations on the morphological diversity and distribution of two siliceous nannoplankton genera Hyalolithus and Petasaria. Micropaleontology, 61(6), 439–455.

[emi16280-bib-0021] Keeling, P.J. , Burki, F. , Wilcox, H.M. , Allam, B. , Allen, E.E. , Amaral‐Zettler, L.A. et al. (2014) The marine microbial eukaryote transcriptome sequencing project (MMETSP): illuminating the functional diversity of eukaryotic life in the oceans through transcriptome sequencing. PLoS Biology, 12(6), e1001889. 24959919 10.1371/journal.pbio.1001889 PMC4068987

[emi16280-bib-0022] Keller, R. , Ziegler, C. & Schneider, D. (2014) When two turn into one: evolution of membrane transporters from half modules. Biological Chemistry, 395(12), 1379–1388. 25296672 10.1515/hsz-2014-0224

[emi16280-bib-0023] Kirkwood, D.S. (1989) Simultaneous determination of selected nutrients in seawater. International Council for the Exploration of the Sea (ICES) Annual Report 29.

[emi16280-bib-0024] Knight, M.J. , Hardy, B.J. , Wheeler, G.L. & Curnow, P. (2022) Computational modelling of diatom silicic acid transporters predicts a conserved fold with implications for their function and evolution. Biochimica et Biophysica Acta, 1865(1), 184056. 36191629 10.1016/j.bbamem.2022.184056

[emi16280-bib-0025] Knight, M.J. , Senior, L. , Nancolas, B. , Ratcliffe, S. & Curnow, P. (2016) Direct evidence of the molecular basis for biological silicon transport. Nature Communications, 7, 11926. 10.1038/ncomms11926 PMC4912633 27305972

[emi16280-bib-0026] Krause, J.W. , Brzezinski, M.A. & Jones, J.L. (2011) Application of low‐level beta counting of Si‐32 for the measurement of silica production rates in aquatic environments. Marine Chemistry, 127(1–4), 40–47.

[emi16280-bib-0027] Krausse, G.L. , Schelske, C.L. & Davis, C.O. (1983) Comparison of three wet‐alkaline methods of digestion of biogenic silica in water. Freshwater Biology, 13, 73–81.

[emi16280-bib-0028] Kumar, S. , Rechav, K. , Kaplan‐Ashiri, I. & Gal, A. (2020) Imaging and quantifying homeostatic levels of intracellular silicon in diatoms. Science Advances, 6(42), eaaz7554. 33067244 10.1126/sciadv.aaz7554 PMC7567585

[emi16280-bib-0029] Langer, G. & Benner, I. (2009) Effect of elevated nitrate concentration on calcification in *Emiliania huxleyi* . Journal of Nannoplankton Research, 30, 77–80.

[emi16280-bib-0030] Langer, G. , Geisen, M. , Baumann, K.H. , Klas, J. , Riebesell, U. , Thoms, S. et al. (2006) Species‐specific responses of calcifying algae to changing seawater carbonate chemistry. Geochemistry Geophysics Geosystems, 7, Q09006.

[emi16280-bib-0031] Langer, G. , Nehrke, G. , Probert, I. , Ly, J. & Ziveri, P. (2009) Strain‐specific responses of *Emiliania huxleyi* to changing seawater carbonate chemistry. Biogeosciences, 6(11), 2637–2646.

[emi16280-bib-0032] Langer, G. , Oetjen, K. & Brenneis, T. (2012) Calcification of *Calcidiscus leptoporus* under nitrogen and phosphorus limitation. Journal of Experimental Marine Biology and Ecology, 413, 131–137.

[emi16280-bib-0033] Langer, G. , Taylor, A.R. , Walker, C.E. , Meyer, E.M. , Ben Joseph, O. , Gal, A. et al. (2021) Role of silicon in the development of complex crystal shapes in coccolithophores. The New Phytologist, 231, 1845–1857. 33483994 10.1111/nph.17230

[emi16280-bib-0034] Leblanc, K. , Hare, C.E. , Feng, Y. , Berg, G.M. , DiTullio, G.R. , Neeley, A. et al. (2009) Distribution of calcifying and silicifying phytoplankton in relation to environmental and biogeochemical parameters during the late stages of the 2005 North East Atlantic Spring Bloom. Biogeosciences, 6(10), 2155–2179.

[emi16280-bib-0035] Likhoshway, Y.V.M.Y. , Sherbakova, T.A. , Petrova, D.P. & Grachev, A.M.A. (2006) Detection of the gene responsible for silicic acid transport in chrysophycean algae. Doklady Biological Sciences, 408, 256–260. 16909993 10.1134/s001249660603015x

[emi16280-bib-0036] Liu, H. , Aris‐Brosou, S. , Probert, I. & de Vargas, C. (2010) A time line of the environmental genetics of the haptophytes. Molecular Biology and Evolution, 27(1), 161–176. 19762334 10.1093/molbev/msp222

[emi16280-bib-0037] Ma, J.F. , Yamaji, N. , Mitani, N. , Tamai, K. , Konishi, S. , Fujiwara, T. et al. (2007) An efflux transporter of silicon in rice. Nature, 448(7150), 209–212. 17625566 10.1038/nature05964

[emi16280-bib-0038] Maldonado, M. (2009) Embryonic development of verongid demosponges supports the independent acquisition of spongin skeletons as an alternative to the siliceous skeleton of sponges. Biological Journal of the Linnean Society, 97(2), 427–447.

[emi16280-bib-0039] Maldonado, M. , Lopez‐Acosta, M. , Beazley, L. , Kenchington, E. , Koutsouveli, V. & Riesgo, A. (2020) Cooperation between passive and active silicon transporters clarifies the ecophysiology and evolution of biosilicification in sponges. Science Advances, 6(28), eaba9322. 32832609 10.1126/sciadv.aba9322 PMC7439455

[emi16280-bib-0040] Marron, A.O. , Alston, M.J. , Heavens, D. , Akam, M. , Caccamo, M. , Holland, P.W. et al. (2013) A family of diatom‐like silicon transporters in the siliceous loricate choanoflagellates. Proceedings of the Biological Sciences, 280(1756), 20122543. 10.1098/rspb.2012.2543 PMC3574361 23407828

[emi16280-bib-0041] Marron, A.O. , Ratcliffe, S. , Wheeler, G.L. , Goldstein, R.E. , King, N. , Not, F. et al. (2016) The evolution of silicon transport in eukaryotes. Molecular Biology and Evolution, 33(12), 3226–3248. 27729397 10.1093/molbev/msw209 PMC5100055

[emi16280-bib-0042] McNair, H.M. , Brzezinski, M.A. , Till, C.P. & Krause, J.W. (2018) Taxon‐specific contributions to silica production in natural diatom assemblages. Limnology and Oceanography, 63(3), 1056–1075. 29937577 10.1002/lno.10754 PMC6007990

[emi16280-bib-0043] Misra, S. , Greaves, M. , Owen, R. , Kerr, J. , Elmore, A.C. & Elderfield, H. (2014) Determination of B/Ca of natural carbonates by HR‐ICP‐MS. Geochemistry Geophysics Geosystems, 15(4), 1617–1628.

[emi16280-bib-0044] Moestrup, O. (1979) Identification by electron‐microscopy of marine nanoplankton from New Zealand, including the description of 4 new species. New Zealand Journal of Botany, 17(1), 61–95.

[emi16280-bib-0045] Ohnemus, D.C. , Krause, J.W. , Brzezinski, M.A. , Collier, J.L. , Baines, S.B. & Twining, B.S. (2018) The chemical form of silicon in marine Synechococcus. Marine Chemistry, 206, 44–51.

[emi16280-bib-0046] Oviedo, A.M. , Langer, G. & Ziveri, P. (2014) Effect of phosphorus limitation on coccolith morphology and element ratios in Mediterranean strains of the coccolithophore *Emiliania huxleyi* . Journal of Experimental Marine Biology and Ecology, 459, 105–113.

[emi16280-bib-0047] Paasche, E. (1980) Silicon content of five marine plankton diatom species measured with a rapid filter method. Limnology and Oceanography., 25(3), 474–480.

[emi16280-bib-0048] Patil, S. , Mohan, R. , Gazi, S. , Shetye, S. & Jafar, S.A. (2015) *Petasaria heterolepis* (Prymnesiaceae) from the southern Indian Ocean. Micropaleontology, 61(3), 171–176.

[emi16280-bib-0049] Pfaffl, M.W. , Horgan, G.W. & Dempfle, L. (2002) Relative expression software tool (REST) for group‐wise comparison and statistical analysis of relative expression results in real‐time PCR. Nucleic Acids Research, 30(9), e36. 11972351 10.1093/nar/30.9.e36 PMC113859

[emi16280-bib-0050] Ratcliffe, S. , Jugdaohsingh, R. , Vivancos, J. , Marron, A. , Deshmukh, R. , Ma, J.F. et al. (2017) Identification of a mammalian silicon transporter. American Journal of Physiology. Cell Physiology, 312(5), C550–C561. 28179233 10.1152/ajpcell.00219.2015 PMC5451523

[emi16280-bib-0051] Sheward, R.M. , Bown, P.R. , Gibbs, S.J. , Poulton, A.J. & O'Dea, S.A. (2017) Wanted: dead and alive! Integrating extant with extinct to transform our understanding of coccolithophore physiology ecology and evolution. Phycologia, 56(4), 171–172.

[emi16280-bib-0052] Shrestha, R.P. & Hildebrand, M. (2015) Evidence for a regulatory role of diatom silicon transporters in cellular silicon responses. Eukaryotic Cell, 14(1), 29–40. 25380754 10.1128/EC.00209-14 PMC4279021

[emi16280-bib-0053] Shrestha, R.P. , Tesson, B. , Norden‐Krichmar, T. , Federowicz, S. , Hildebrand, M. & Allen, A.E. (2012) Whole transcriptome analysis of the silicon response of the diatom *Thalassiosira pseudonana* . BMC Genomics, 13, 499. 22994549 10.1186/1471-2164-13-499 PMC3478156

[emi16280-bib-0054] Silkin, V. , Pautova, L. , Giordano, M. , Kravchishina, M. & Artemiev, V. (2020) Interannual variability of *Emiliania huxleyi* blooms in the Barents Sea: in situ data 2014‐2018. Marine Pollution Bulletin, 158, 111392. 32753178 10.1016/j.marpolbul.2020.111392

[emi16280-bib-0055] Skampa, E. , Triantaphyllou, M.V. , Dimiza, M.D. , Gogou, A. , Malinverno, E. , Stavrakakis, S. et al. (2020) Coccolithophore export in three deep‐sea sites of the Aegean and Ionian seas (eastern Mediterranean): biogeographical patterns and biogenic carbonate fluxes. Deep‐Sea Research Part Ii‐Topical Studies in Oceanography, 171, 104690.

[emi16280-bib-0056] Smith, S.R. , Gle, C. , Abbriano, R.M. , Traller, J.C. , Davis, A. , Trentacoste, E. et al. (2016) Transcript level coordination of carbon pathways during silicon starvation‐induced lipid accumulation in the diatom *Thalassiosira pseudonana* . The New Phytologist, 210(3), 890–904. 26844818 10.1111/nph.13843 PMC5067629

[emi16280-bib-0057] Smyth, T.J. , Fishwick, J.R. , Al‐Moosawi, L. , Cummings, D.G. , Harris, C. , Kitidis, V. et al. (2010) A broad spatio‐temporal view of the Western English Channel observatory. Journal of Plankton Research, 32(5), 585–601.

[emi16280-bib-0058] Taylor, A.R. , Brownlee, C. & Wheeler, G. (2017) Coccolithophore cell biology: chalking up Progress. Annual Review of Marine Science, 9(9), 283–310. 10.1146/annurev-marine-122414-034032 27814031

[emi16280-bib-0059] Thamatrakoln, K. , Alverson, A.J. & Hildebrand, M. (2006) Comparative sequence analysis of diatom silicon transporters: toward a mechanistic model of silicon transport. Journal of Phycology, 42(4), 822–834.

[emi16280-bib-0060] Thamatrakoln, K. & Hildebrand, M. (2007) Analysis of *Thalassiosira pseudonana* silicon transporters indicates distinct regulatory levels and transport activity through the cell cycle. Eukaryotic Cell, 6(2), 271–279. 17172435 10.1128/EC.00235-06 PMC1797941

[emi16280-bib-0061] Thamatrakoln, K. & Hildebrand, M. (2008) Silicon uptake in diatoms revisited: a model for saturable and nonsaturable uptake kinetics and the role of silicon transporters. Plant Physiology, 146(3), 1397–1407. 18162598 10.1104/pp.107.107094 PMC2259041

[emi16280-bib-0062] Treguer, P.J. (2014) The Southern Ocean silica cycle. Comptes Rendus Geoscience, 346(11–12), 279–286.

[emi16280-bib-0063] Villar, E. , Vannier, T. , Vernette, C. , Lescot, M. , Cuenca, M. , Alexandre, A. et al. (2018) The Ocean Gene Atlas: exploring the biogeography of plankton genes online. Nucleic Acids Research, 46(W1), W289–W295. 29788376 10.1093/nar/gky376 PMC6030836

[emi16280-bib-0064] Walker, C.E. , Taylor, A.R. , Langer, G. , Durak, G.M. , Heath, S. , Probert, I. et al. (2018) The requirement for calcification differs between ecologically important coccolithophore species. New Phytologist, 220(1), 147–162. 29916209 10.1111/nph.15272 PMC6175242

[emi16280-bib-0065] Yool, A. & Tyrrell, T. (2003) Role of diatoms in regulating the ocean's silicon cycle. Global Biogeochemical Cycles, 17(4), 1103.

[emi16280-bib-0066] Yoshida, M. , Noel, M.H. , Nakayama, T. , Naganuma, T. & Inouye, I. (2006) A haptophyte bearing siliceous scales: ultrastructure and phylogenetic position of *Hyalolithus neolepis* gen. et sp nov (Prymnesiophyceae, Haptophyta). Protist, 157(2), 213–234. 16647294 10.1016/j.protis.2006.02.004

[emi16280-bib-0067] Ziveri, P. , de Bernardi, B. , Baumann, K.H. , Stoll, H.M. & Mortyn, P.G. (2007) Sinking of coccolith carbonate and potential contribution to organic carbon ballasting in the deep ocean. Deep‐Sea Research Part Ii‐Topical Studies in Oceanography, 54(5–7), 659–675.

[emi16280-bib-0068] Zlatogursky, V.V. (2016) There and back again: parallel evolution of cell coverings in centrohelid heliozoans. Protist, 167(1), 51–66. 26828628 10.1016/j.protis.2015.12.002

