# Supplementary material for: Characterization of the molecular mechanisms of silicon uptake in coccolithophores
Source: Environ Microbiol. 2022 Nov 22;25(2):315–30. doi: 10.1111/1462-2920.16280 (PMC10098502; doi:10.1111/1462-2920.16280)
Supplement: Supplementary file 1 — Appendix S1. Supporting Information. [file EMI-25-315-s001.docx]

**Supplementary data**

**Supplementary Figures 1-6**

**Supplementary Tables 1-5**

**
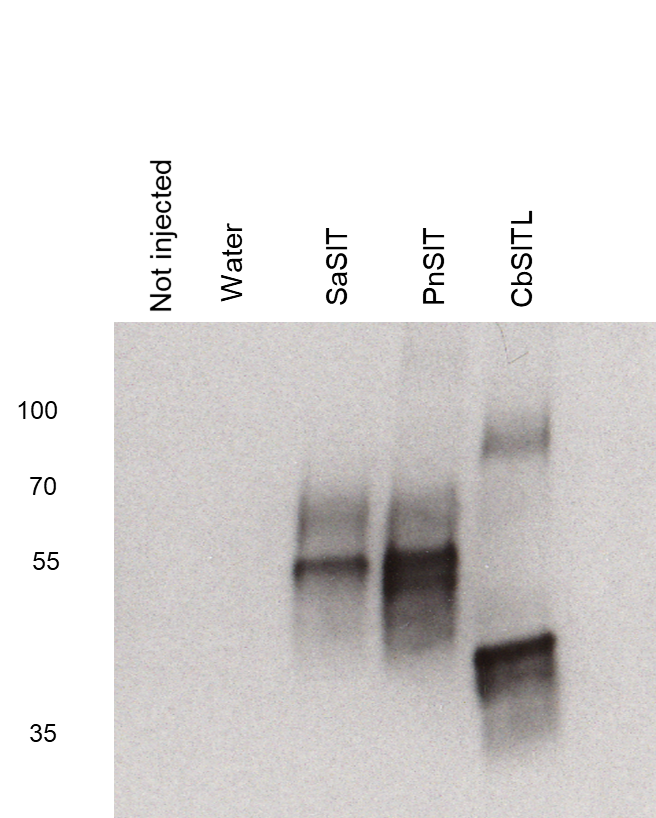
**

**Supplementary Figure 1: Expression of CbSITL in Xenopus oocytes.** Western blot showing expression of CbSITL in *Xenopus* oocytes. A major band can be observed at approximately 45 kD, with a minor band at 90 kD that potentially represents dimer formation. The primary antibody targeted the C-terminal V5-epitope tag. Extracts from control (uninjected) and water-injected oocytes show no reactivity. Lanes marked SaSIT and PnSIT were not involved in this study.


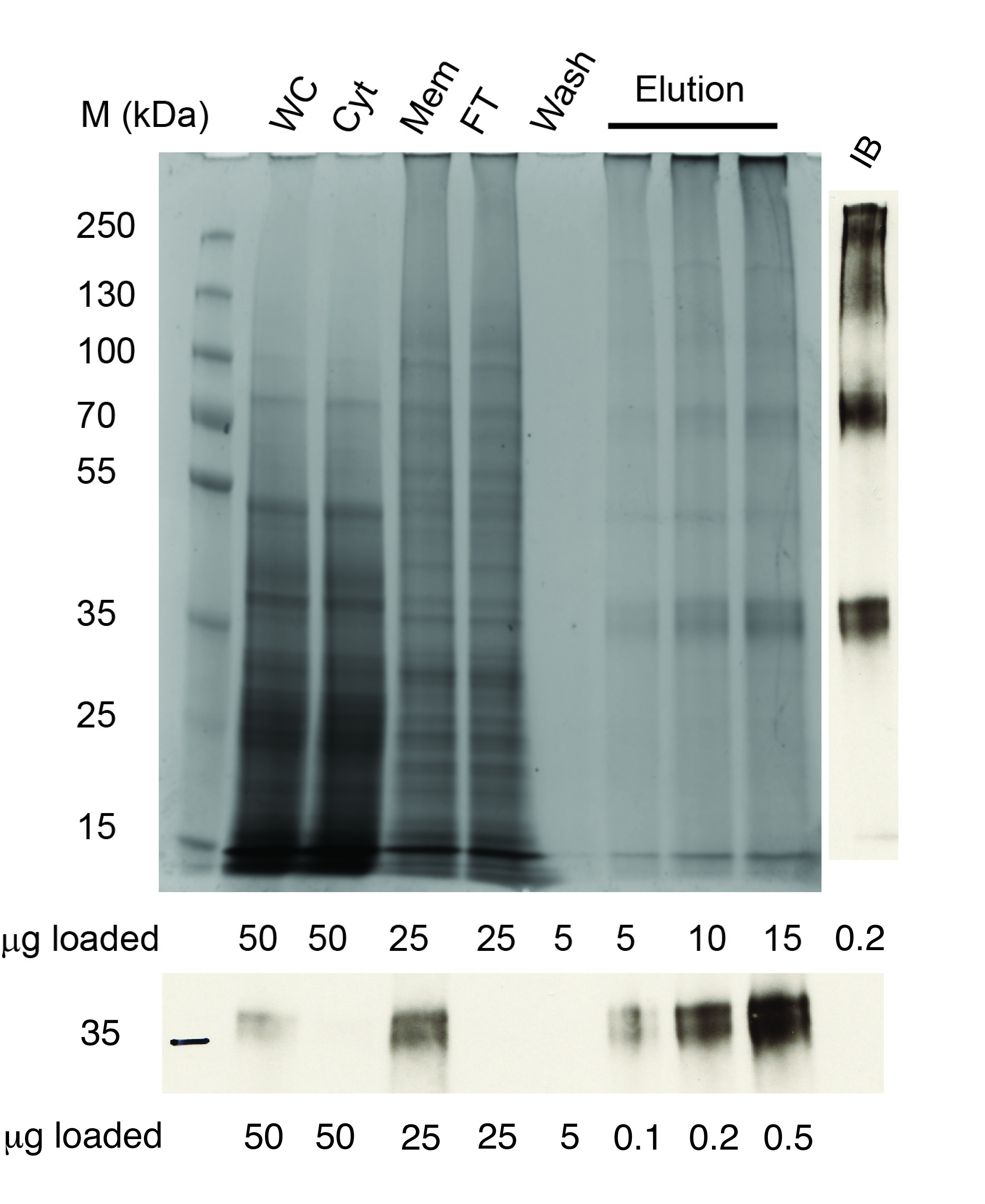


**Supplementary Figure 2: Expression of recombinant *CbSITL* in *S. cerevisiae*.** An image of a Coomassie-stained SDS-PAGE gel showing the expression of *CbSITL* in yeast. CbSITL runs as a doublet close to the expected molecular weight (40.7kD). Labels correspond to cell fractions (WC, whole-cell lysate; Cyt, cytoplasm; Mem, membranes) or purification fractions (FT, column flow-through or unbound; Wash, eluate from column washing; Elution, bound protein eluted with imidazole). The right panel shows a Western blot (IB) using an anti-V5 primary antibody, confirming the identity of the major bands as *CbSITL*, note the higher molecular weight band at c. 75 kD, which may represent dimer formation. The bottom panel is an immunoblot of the different fractions, showing that CbSITL is found primarily in the membrane fraction, binds tightly to the affinity column (absent in flow-through) and is substantially enriched in the eluate.

**
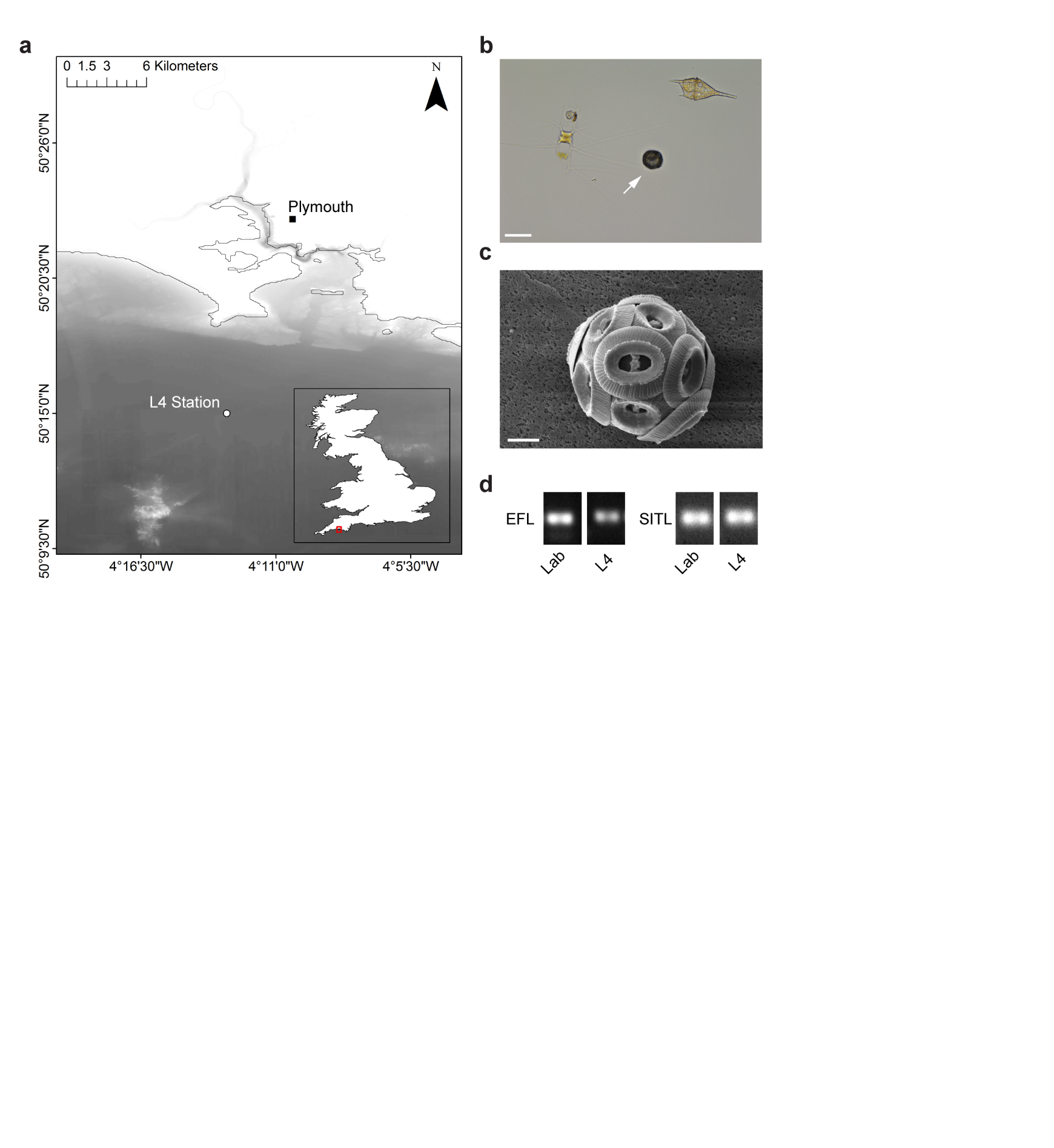
**

**Supplementary Figure 3:** **Expression of SITLs in natural coccolithophore populations. A)** Position of L4 station where plankton sampling took place. **B**) Light microscopy of L4 plankton samples demonstrating the presence of *C. braarudii* (arrowed). Scale bar = 20 μm. **C**) SEM image of *C. braarudii* from the sample shown in (B). Scale bar = 5 μm. **D**) RT-PCR of the reference gene *EFL* and *CbSITL* from a laboratory culture of *C. braarudii* (lab) and from an environmental sample (L4). Positive sequences were successfully amplified from environmental RNA extracted from the L4 sample, confirming the presence and expression of *CbSITL* in a natural population of *C. braarudii*.

**
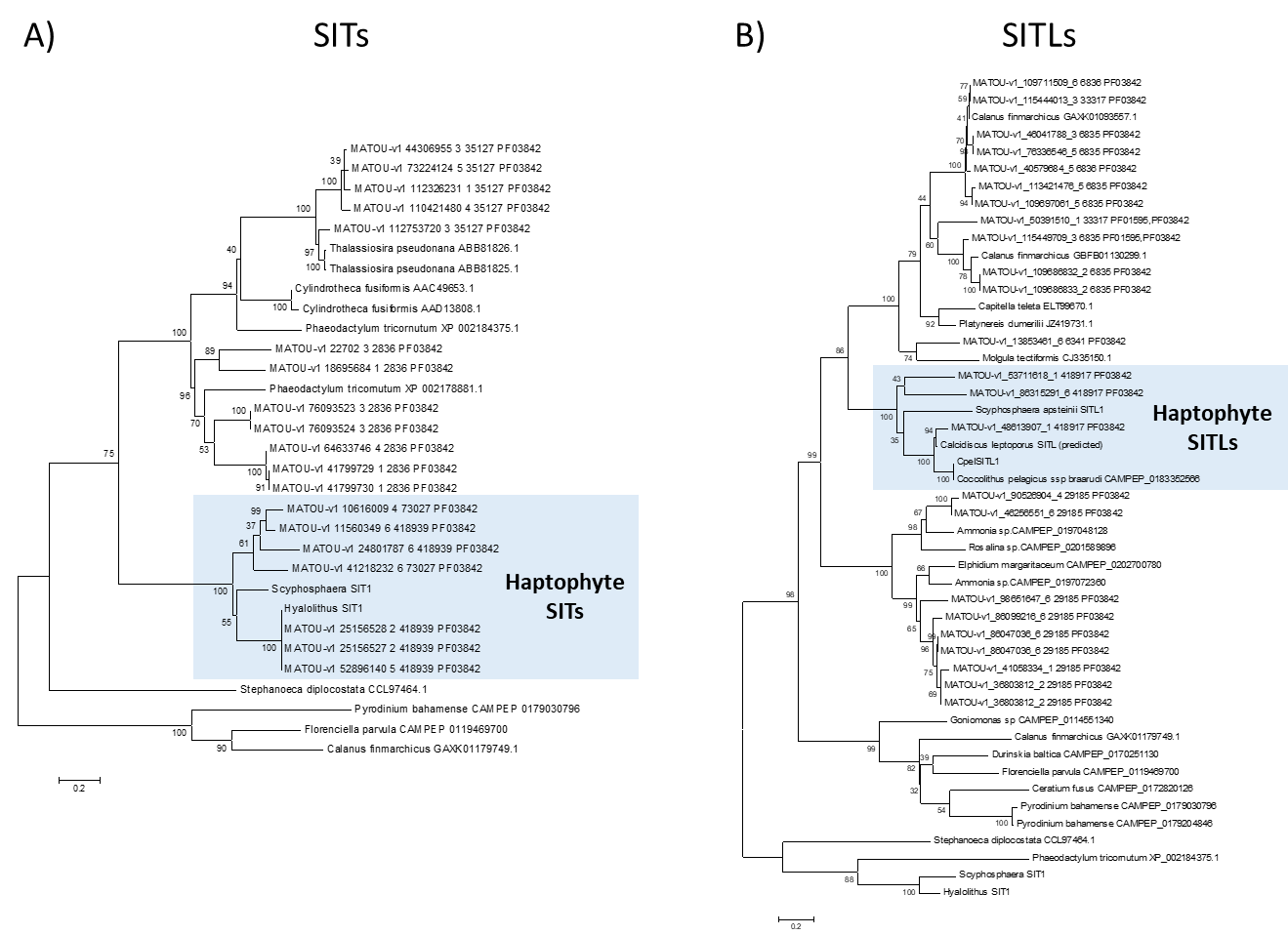
**

**Supplementary Figure 4: Haptophyte Si transporters identified in the TARA Oceans dataset. A)** Phylogenetic tree of Group 1 SITs showing the relationship between SITs from cultured organisms and additional TARA Ocean Unigene accessions. The well supported phylogenetic clade including *P. neolepis* and *S. apsteinii* likely represents haptophyte SITs and suggests that many other haptophytes possess 10-TM SITs. The tree was constructed using the maximum likelihood method, using an alignment of 154 amino acids. Numbers above nodes represent bootstrap values (100 bootstraps were performed). The tree was rooted with Group 1 SITL sequences. Accessions *S. apsteinii* SIT1 CAMPEP_0119335116, *Hyalolithus* (now *P. neolepis*) SIT1 KP793098, *C. leptoporus* SITL1 KR677451, *S. apsteinii* SITL1 CAMPEP_0119299848. **B**) Phylogenetic tree of Group 1 SITLs showing the relationship between SITLs from cultured organisms and TARA Ocean Unigene accessions. Note that many accessions group with copepods and foraminifera. The well supported phylogenetic clade including coccolithophore SITLs likely represents additional haptophyte SITLs. The tree was constructed using the maximum likelihood method. Numbers above nodes represent bootstrap values (100 bootstraps were performed). The tree was rooted with Group 1 SIT sequences (N-terminus).

**Supplementary Figure 5:** Distribution of haptophyte Si transporters in the TARA Oceans dataset. A) The maps indicate the abundance of reads belonging to either the haptophyte 10-TM SITs (top) or haptophyte 5-TM SITLs (bottom) found at the surface ocean (SRF) or the deep chlorophyll maximum (DCM) samples. The reads for haptophyte SITs include those for the silicifying haptophyte *P. neolepis.* **B)** DSi concentrations from sampling stations where haptophyte Si transporters were identified. TARA Ocean Unigene accessions (MATOU) belonging to haptophyte SIT and SITL are shown, with SITs sub-divided in those showing very high similarity to *P. neolepsis* and other haptophyte SITs.





**Supplemental Figure 6**: SEM micrographs of coccolithophores at the start (left column) and end (right column) of an 8 d DSi depletion experiment (see Figure 4). Each species started with approximately 5 µmol/L Si. Species are (a) *Emiliania huxleyi* (no SITL), (b) *Gephyrocapsa oceanica* (no SITL), (c) *Calcidiscus leptoporus* (SITL), (d) *Coccolithus braarudii* (SITL), and (e) *Scyphosphaera apsteinii* (SIT/SITL). For all species, there was no change in coccolith morphology after 8 days.

| Gene Name | Full Name | Target | Sequence ID | Primer Name | Primer Sequence |
| --- | --- | --- | --- | --- | --- |
| *SITL* | SITL Si Transporter | Query | CAMNT_0025525031 | CbrSITL_F | CGCTGGCATGAATCAAGGTG |
|  |  |  |  | CbrSITL_R | CATATTCCTCCGCACGTCGT |
| *EFL* | Elongation Factor | Reference | CAMNT_0025499507 | CbrEFL_F | GTGCACCACCAAGGAGTTCT |
|  |  |  |  | CbrEFL_R | GTGGTTGCCCTTTTGGATGG |
| *RPS1* | Ribosomal Protein S1 | Reference | CAMNT_0025558139 | CbrRPS1_F | GCGTCGGAGAAGACAGACTC |
|  |  |  |  | CbrRPS1_R | GGGAGACATGCTCAAGAACCA |

**Supplementary Table 1: Primer details for qRT-PCR analysis of *CbSITL* expression**

| Treatment | Time | Cell density | Counts | Si uptake | Growth rate | Detection limit |  |
| --- | --- | --- | --- | --- | --- | --- | --- |
|  | (hr) | (cells mL^-1^) | dpm | µmol L^-1^ d^-1^ | d^-1^ | pmol cell^-1^ d^-1^ |  |
| Live | 4 | 6,300  ± 240 | 11.0  ± 0.5 | 0.08  ± 0.004 | 0.59 | 0.022  ± 0.007 | |
| Dead | 4 | 3,900  ± 970 | 12.1  ± 4.4 | 0.09  ± 0.027 | 0.07 |  | |
| Ge | 4 | 6,500  ± 400 | 10.5  ± 2.6 | 0.06  ± 0.016 | 0.18 |  | |
|  |  |  |  |  |  |  | |
| Live | 24 | 10,400  ± 1,200 | 18.2  ± 6.4 | 0.02  ± 0.008 |  | 0.006  ± 0.003 | |
| Dead | 24 | 4,200  ± 470 | 18.7  ± 10.3 | 0.02  ± 0.013 |  |  | |
| Ge | 24 | 7,600  ± 1,000 | 21.5  ± 7.3 | 0.03  ± 0.009 |  |  | |

**Supplementary Table 2: Measurement of ^32^Si uptake by *C. braarudii.*** Results are the mean of 3 experimental replicates ± s.d.

| Treatment | Time | Cell density | Counts | Si uptake | Growth rate | Detection limit |
| --- | --- | --- | --- | --- | --- | --- |
|  | (hr) | (cells mL^-1^) | dpm | µmol L^-1^ d^-1^ | d^-1^ | pmol cell^-1^ d^-1^ |
| Live | 4 | 44,000  ± 5,800 | 10.8  ± 2.5 | 0.08  ± 0.02 | 0.87 | 0.0018 |
| Dead | 4 | 18,000  ± 7,400 | 11.7  ± 1.8 | 0.09  ± 0.02 | -1.89 |  |
| Ge | 4 | 45,000  ± 6,400 | 11.3  ± 1.9 | 0.08  ± 0.01 | 0.57 |  |
|  |  |  |  |  |  |  |
| Live | 24 | 92,000  ± 5,000 | 13.8  ± 0.9 | 0.02  ± 0.001 |  | 0.0054 |
| Dead | 24 | 3,800  ± 1,700 | 15.4  ± 6.3 | 0.02  ± 0.01 |  |  |
| Ge | 24 | 73,000  ± 9,900 | 12.9  ± 0.7 | 0.02  ± 0.001 |  |  |

**Supplementary Table 3: Measurement of ^32^Si uptake by *E. huxleyi.*** Results are the mean of 3 experimental replicates ± s.d.

| Species | Day 0 | Day 1 | Day 2 | Day 3 | Day 4 | Day 6 | Day 8 |
| --- | --- | --- | --- | --- | --- | --- | --- |
| *T. weissflogii* | 0.10  ± 0.18 | 4.10  ± 7.05 | 5.99  ± 9.96 | 3.30  ± 5.28 | 4.41  ± 7.20 | 3.61  ± 5.74 | 0.30  ± 0.34 |
| *P. neolepis* | 4.19  ± 2.87 | 5.47  ± 3.77 | 9.09  ± 0.89 | 11.04  ± 1.79 | 5.80  ± 1.26 | 4.23  ± 0.58 | 4.28  ± 0.87 |
| *E. huxleyi* | 0.00  ± 0.00 | 0.01  ± 0.01 | 0.03  ± 0.04 | 0.002  ± 0.003 | 0.00  ± 0.00 | 0.001  ± 0.000 | 0.00  ± 0.00 |
| *G. oceanica* | 0.00  ± 0.00 | 0.07  ± 0.12 | 0.004  ± 0.008 | 0.03  ± 0.01 | 0.01  ± 0.01 | 0.003  ± 0.005 | 0.002  ± 0.002 |
| *S. apsteinii* | 0.23  ± 0.13 | 0.10  ± 0.18 | 0.15  ± 0.16 | 0.00  ± 0.00 | 0.00  ± 0.00 | 0.03  ± 0.03 | 0.04  ± 0.04 |
| *C. braarudii* | 0.09  ± 0.06 | 0.25  ± 0.20 | 0.07  ± 0.05 | 0.07  ± 0.05 | 0.02  ± 0.03 | 0.03  ± 0.06 | 0.02  ± 0.01 |
| *C. leptoporus* | 0.09  ± 0.02 | 0.00  ± 0.00 | 0.03  ± 0.06 | 0.08  ± 0.06 | 0.002  ± 0.004 | 0.01  ± 0.01 | 0.001  ± 0.001 |

**Supplementary Table 4: Si incorporation into biogenic structures (BSi).** BSi in pmol cell^-1^ ± standard deviation. BSi was determined by filtering cells, using a modified alkaline digestion, centrifuging, and running the supernatant through an autoanalyzer. Coccolithophore measurements could not be discerned from the background media.

| Species | C At% | Ca At% | O At% | Na At% | Mg At% | Si At% | n  cells | n  coccoliths |
| --- | --- | --- | --- | --- | --- | --- | --- | --- |
| *E.huxleyi*  CCMP 1516 | 58.53  ±7.01 | 7.11  ±1.90 | 34.13  ±5.46 | 0.05  ±0.04 | 0.03  ±0.02 | 0.15  ±0.26 | 6 | 20 |
| *E.huxleyi*  PML B92/11 | 65.90  ±3.80 | 5.22  ±0.85 | 28.74  ±3.10 | 0.05  ±0.06 | 0.03  ±0.02 | 0.00  ±0.00 | 4 | 10 |
| *G.oceanica*  RCC 1303 | 35.51  ±6.50 | 13.84  ±2.22 | 50.53  ±5.09 | 0.06  ±0.02 | 0.01  ±0.01 | 0.04  ±0.02 | 5 | 18 |
| *C.braarudii*  PLY 182G | 25.93  ±4.43 | 17.55  ±3.17 | 56.45  ±3.37 | 0.06  ±0.03 | 0.01  ±0.01 | 0.03  ±0.03 | 8 | 26 |
| *C.leptoporus*  RCC 1130 | 30.73  ±8.46 | 15.85  ±3.50 | 53.31  ±6.08 | 0.06  ±0.03 | 0.01  ±0.02 | 0.04  ±0.02 | 8 | 28 |
| *S.apsteinii* M  RCC 1456 | 26.21  ±3.40 | 19.40  ±3.69 | 53.95  ±4.67 | 0.11  ±0.03 | 0.02  ±0.01 | 0.03  ±0.03 | 7 | 21 |
| *S.apsteinii* L  RCC 1456 | 27.28  ±5.01 | 18.06  ±3.11 | 54.14  ±3.63 | 0.05  ±0.03 | 0.01  ±0.01 | 0.01  ±0.02 | 7 | 18 |
| *C.leptoporus* (N) RCC 1130 | 76.08  ±4.67 | 3.77  ±4.05 | 22.51  ±10.76 | 0.15  ±0.15 | 0.04  ±0.04 | 0.08  ±0.16 | 8 | 29 |
| *C.braarudii* (N)  RCC 3777 | 64.44  ±5.98 | 6.14  ±1.69 | 29.13  ±4.29 | 0.14  ±0.04 | 0.02  ±0.01 | 0.05  ±0.02 | 7 | 23 |
| *S.pulchra* (N)  RCC 1461 | 50.37  ±9.75 | 10.35  ±3.08 | 38.69  ±6.55 | 0.12  ±0.05 | 0.28  ±0.10 | 0.07  ±0.04 | 6 | 16 |
| *Calyptrosphaera sp.* | 50.52  ±6.22 | 10.27  ±3.73 | 40.15  ±9.70 | 0.61  ±0.25 | 0.53  ±0.29 | 0.07  ±0.04 | 5 | 17 |
| *P.neolepis*  RCC 1453 | 75.00  ±4.52 | 0.00  ±0.00 | 21.42  ±3.53 | 0.14  ±0.05 | 0.05  ±0.01 | 3.44  ±0.98 | 3 | 9 |

**Supplementary Table 5:** Elemental analysis of mineralized scales of coccolithophores and a silicifying haptophyte (*P.neolepis*) using Energy-Dispersive X-ray Spectroscopy (EDS). Average concentrations of the elements in atomic % (At%) ± standard deviation. Species were diploid (2N) unless otherwise stated (N). L= Lopadolith, M = Murolith.
